# Efficacy and safety of behavioural activation on depression in people with co-occurring non-communicable diseases: systematic review and meta-analysis

**DOI:** 10.1192/bjo.2024.870

**Published:** 2025-03-24

**Authors:** Engida Yisma, Kuda Muyambi, Sandra Walsh, Shwikar Othman, Richard Gray, Kuan Liung Tan, Mary Steen, Martin Jones

**Affiliations:** Department of Rural Health, Allied Health & Human Performance, University of South Australia, Adelaide, Australia; IIMPACT in Health, University of South Australia, Adelaide, Australia; UniSA Clinical & Health Sciences, University of South Australia, Adelaide, Australia; School of Nursing and Midwifery, La Trobe University, Melbourne, Australia; Department Nursing, Faculty of Health Sciences, Curtin University, Perth, Australia; School of Nursing and Midwifery, Edith Cowan University South West Campus, Bunbury, Australia

**Keywords:** Behavioural activation, depression, effectiveness, meta-analysis, non-communicable diseases

## Abstract

**Background:**

People with non-communicable diseases (NCDs) have a higher prevalence of comorbid depression than the general population. While previous research has shown that behavioural activation is effective for general depression, its efficacy and safety in treating depression associated with NCDs remains unclear.

**Aims:**

To compare the efficacy and safety of behavioural activation against comparators in reducing depression symptoms in people with NCDs.

**Method:**

We searched six databases from inception until 30 March 2023 (updated 23 September 2024) for randomised controlled trials (RCTs) comparing behavioural activation with comparators for depression in people with NCDs. Risk of bias was assessed using the Cochrane Collaboration’s ‘risk-of-bias 2 tool’. We calculated a random-effects, inverse-variance weighting meta-analysis.

**Results:**

Of the 21 386 initial studies, 12 RCTs (with 2144 patients) comparing behavioural activation with any comparator on treatment outcomes for depression with comorbid NCD met the inclusion criteria. Six studies rated as low risk of bias. For short-term follow-ups (up to 6 months), meta-analysis showed behavioural activation had little effect on depression symptom improvement in people with NCDs (Hedges’ *g* = −0.24; 95% CI, −0.62 to 0.15), compared to comparators, with high heterogeneity (*I* ^2^ = 91.91%). Of the 12 included studies, three RCTs provided data on adverse events occurring during the trial.

**Conclusions:**

Evidence from this systematic review is not sufficient to draw clear conclusions about the efficacy and safety of behavioural activation for reducing depression symptoms in people with NCDs. Future reviews need to include more high-quality, well-designed RCTs to better understand the potential benefits of behavioural activation for comorbid depression.

Depressive disorders were the second highest contributor to the burden of disease in terms of years of life lived with a disability worldwide in 2019.^1^ Approximately 280 million people worldwide were affected by depressive disorders in 2019, with the prevalence rates being higher in females than males.^1^ A 2014 meta-analysis by Cuijpers et al^2^ examined 293 observational studies with 1 813 733 participants from healthy community samples and patient groups, including those with heart disease, cancer, kidney disease, diabetes, chronic obstructive pulmonary disease and other somatic illnesses. They found a 52% higher mortality risk among depressed participants compared to non-depressed participants, with minimal variation in risk across groups.

It has been demonstrated that depression is a significant complication of several long-term conditions.^3^ The positive association between depression and non-communicable diseases (NCDs) is well documented.^4,5^ The relationship is bidirectional, with each condition serving as a risk factor for the other.^6^ Depression frequently co-occurs in four major types of NCDs (also known as the ‘Big Four’): cardiovascular diseases, diabetes, cancer and chronic respiratory diseases.^7^ For example, depression is a common precursor of all types of stroke.^8^ Moreover, depressive symptoms are frequently encountered in patients with cancer^9^ and people diagnosed with diabetes.^4^

Comorbid depression exacerbates the symptoms and outcomes of NCDs. People affected by both conditions experience diminished physical and social functioning, reduced productivity, impaired recovery and quality of life and increased use of healthcare services.^10^ Having multiple NCDs leads to higher mortality than a single NCD^11^ and imposes a substantial financial burden on people, households and national economies.^12^ This major public health challenge has led the United Nations to direct global attention and efforts toward addressing these prevalent long-term health conditions.^13^

Treating depression is important to improve the overall management and treatment of NCDs and improve long-term outcomes for affected people.^14^ Effective treatment of depression is also essential for improved health, quality of life and economic outcomes for affected adults. Pharmacological and psychological therapies, alone or in combination, are the standard of care for the treatment of mild-to-moderate depression.^15^ However, access to psychiatric and advanced psychological care, such as cognitive–behavioural therapy (CBT), can often be problematic owing to shortages of trained healthcare workers.

## Behavioural activation as a candidate treatment for comorbid depression

Behavioural activation, a component of CBT, has been used for decades as the ‘behavioural’ component of CBT or as stand-alone treatment for depression.^16^ The aim of behavioural activation is to reverse the cycle of depression by monitoring mood and increasing engagement in valued activities.^17^ Behavioural activation is easy to deliver and could be a candidate psychological intervention for individuals with depression. Behavioural activation supports the person to engage in meaningful activities and teaches skills to notice changes in mood and its relationship with these activities. Mastery of these activities provides fulfilment and reward. The aim of therapy is to help the patient schedule activity that is inherently rewarding. This engagement with rewarding activity may be particularly important for people with NCDs who experience diminished physical capability.

A 2020 systematic review by Uphoff et al^18^ included randomised controlled trials (RCTs) that enrolled participants with a clinical diagnosis of depression to examine the effectiveness of behavioural activation in treating depression symptoms among adults with NCDs. The review search identified only two relevant studies from the USA, involving a total of 101 stroke survivors and 80 breast cancer patients. The authors concluded there was insufficient evidence to confirm the efficacy of behavioural activation as a treatment for depression in adults with comorbid NCDs.

In this systematic review, we aimed to include RCTs involving participants with a confirmed diagnosis of depression, assessed through a standardised measure such as a diagnostic interview or validated self-report questionnaire. By having broader inclusion criteria, this review will provide a more comprehensive synthesis of the available evidence on the efficacy and safety of behavioural activation on depression in people with any comorbid NCD. The primary objective of this study is to compare the efficacy of behavioural activation against comparators in reducing depressive symptoms in people with NCDs, while the secondary objective is to examine the safety of behavioural activation compared to comparators in people with any comorbid NCD.

## Method

We conducted a systematic review and meta-analysis of RCTs reporting on the efficacy and safety of behavioural activation on depression in adults with NCDs. The procedures for the review were prespecified in a registered protocol (Open Science Framework (OSF): https://osf.io/7tsa8) and a statistical analysis plan was finalised before any analyses were undertaken. We followed the 2020 PRISMA reporting guidelines for systematic reviews and meta-analyses^19^ (Supplementary material I available at https://doi.org/10.1192/bjo.2024.870).

### Type of studies

This review included RCTs involving participants aged 18 and older with depression and comorbid NCDs such as cancer, cardiovascular disease (e.g. coronary heart disease and stroke), chronic respiratory conditions and diabetes. We focused on studies involving behavioural activation as the primary treatment based on any type of delivery mode, including face-to-face or online, individual or group sessions. Peer-reviewed publications were considered and only studies published in English were included.

### Intervention

We included RCTs that assessed treatment approaches for depression in people with NCDs explicitly labelled as ‘behavioural activation’. Moreover, we considered RCTs that described the interventions utilising the core components of behavioural activation for depression, such as mood monitoring and activity scheduling.

### Comparator

Any comparator intervention including but not limited to treatment as usual, other psychological treatment and antidepressant medication were considered.

### Outcome measures

The outcome of interest was depression as determined by any standardised depression scales, including but not limited to the Hospital Anxiety and Depression Scale (HADS) and the Patient Health Questionnaire-9 (PHQ-9). When a trial included multiple depression measures for the primary outcome, only one scale was chosen based on the most commonly used scale.^20^

### Search strategy

We searched six databases (MEDLINE, Embase, Emcare, PsycINFO, CINAHL and the Cochrane Library), as well as trial registries (the World Health Organization International Clinical Trials Registry (ICTRP), the Australian New Zealand Clinical Trials Registry (ANZCTR) and ClinicalTrials.gov). The search was conducted from the inception of each database up to 30 March 2023 (updated 23 September 2024). We also performed hand searching by examining reference lists of included studies and relevant reviews to identify additional trials missed by electronic searches. The detailed search strategy used in this review can be found in Supplementary material II (Tables S1–S6).

### Study selection

We used Covidence (for Windows: Covidence, Melbourne, Australia; https://www.covidence.org), a web-based review management software to facilitate and manage the study selection process. Five review authors (E.Y., K.M., S.O., S.W. and M.J.) independently screened the titles and/or abstracts of all publications obtained through the search strategy. We then obtained full articles for all RCTs, and the same five review authors (E.Y., K.M., S.O., S.W. and M.J.) assessed the full texts according to criteria relating to study, participant, intervention and outcome characteristics. We discussed any disagreements with a third review author (chosen from E.Y., K.M., S.O., S.W. and M.J.) to reach consensus. We recorded the reasons for excluding studies that did not meet the inclusion criteria.

### Data extraction

We used data extraction forms to retrieve information from the studies incorporated in this review. The data were extracted on 15 January 2024 (for the updated search, no data extraction was done as no study was eligible for data extraction). E.Y., K.M., S.O., S.W. and M.J. independently extracted data from included studies. Any discrepancies among these authors were resolved through discussion with an additional member of the review team (chosen from E.Y., K.M., S.O., S.W. and M.J.). The following information were extracted from each included trial: (a) trial details, such as authors’ names, publication year, study design, follow-up duration, outcome measures (type and timepoints) and details of intervention (type, frequency, etc.); and (b) statistical data (mean, standard deviation and other effect estimate measures) for the primary outcome. To categorise treatment time points for post-treatment outcomes as well as outcomes at each reported follow-up point, we used the cut-offs described by Uphoff et al^21^ defining short term as up to 6 months post-treatment, medium term as 7–12 months post-treatment and long term as more than 12 months post-treatment.

### Assessment of risk of bias in included studies

Two review authors (E.Y. and M.J.) completed the risk of bias assessment. The risk of bias was determined using the Cochrane Collaboration’s ‘risk-of-bias 2 tool’.^22^ The tool considers the following domains: (i) risk of bias arising from the randomisation process, including allocation and randomisation (ii) risk of bias because of deviations from the intended interventions, including blinding of participants and people delivering the interventions, (iii) risk of bias because of missing outcome data, (iv) risk of bias in measurement of the outcome, including blinding of outcome assessors, and (v) risk of bias in the selection of reported results.

### Data synthesis and analysis

We conducted both a narrative synthesis and a meta-analysis of the findings from the included studies. For each comparison between behavioural activation and a comparator, we calculated effect sizes as Hedges’ *g*, which indicates the difference between the two groups at each follow-up point. When studies did not report means and standard deviations, we calculated the effect size using dichotomous outcomes. If these were unavailable, we employed alternative statistics (such as *t*-values or *F*-values) to calculate the effect size.

We used a random-effects model for the meta-analysis to account for differences between studies. Inverse-variance weighting was used to pool effect sizes across the studies in the meta-analysis. The meta-analysis synthesised data on short-term, medium-term and long-term efficacy of behavioural activation compared to the comparator. The summary effect size was reported as a Hedges’ *g* with a 95% confidence interval. We also conducted a meta-analysis by NCD type for short-term outcomes (up to 6 months) to compare findings across similar studies.

To examine the heterogeneity of effect sizes, we computed the *I* ^2^-statistic. The *I* ^2^-statistic serves as an indicator of heterogeneity, expressed as a percentage, with values ranging from 0%, indicating no observed heterogeneity, to 25% denoting low, 50% moderate and 75% high heterogeneity.^23^

### Subgroup analyses

As part of our *a priori* analyses plan, we conducted subgroup analyses to understand the possible sources of heterogeneity associated with pooled estimate of the association between behavioural activation and depression in people with NCDs, based on different parameters. These included types of control group, diagnosis (clinical interviews versus scoring above a cut-off), risk of bias, number of behavioural activation sessions provided, types of intervention, format of intervention, types of study design (individually RCTs and cluster RCTs), types of NCD and types of depression measure scales used. The subcategories were decided based on a previous study^24^ and expert opinion. All statistical analyses for the meta-analysis were performed using STATA/SE for Windows version 18.0 (Stata Corporation, College Station, Texas, USA).

### Patient and public involvement

There were no patients or public involvement in any aspect of this research.

## Results

### Results of the searches

**Fig. 1:**
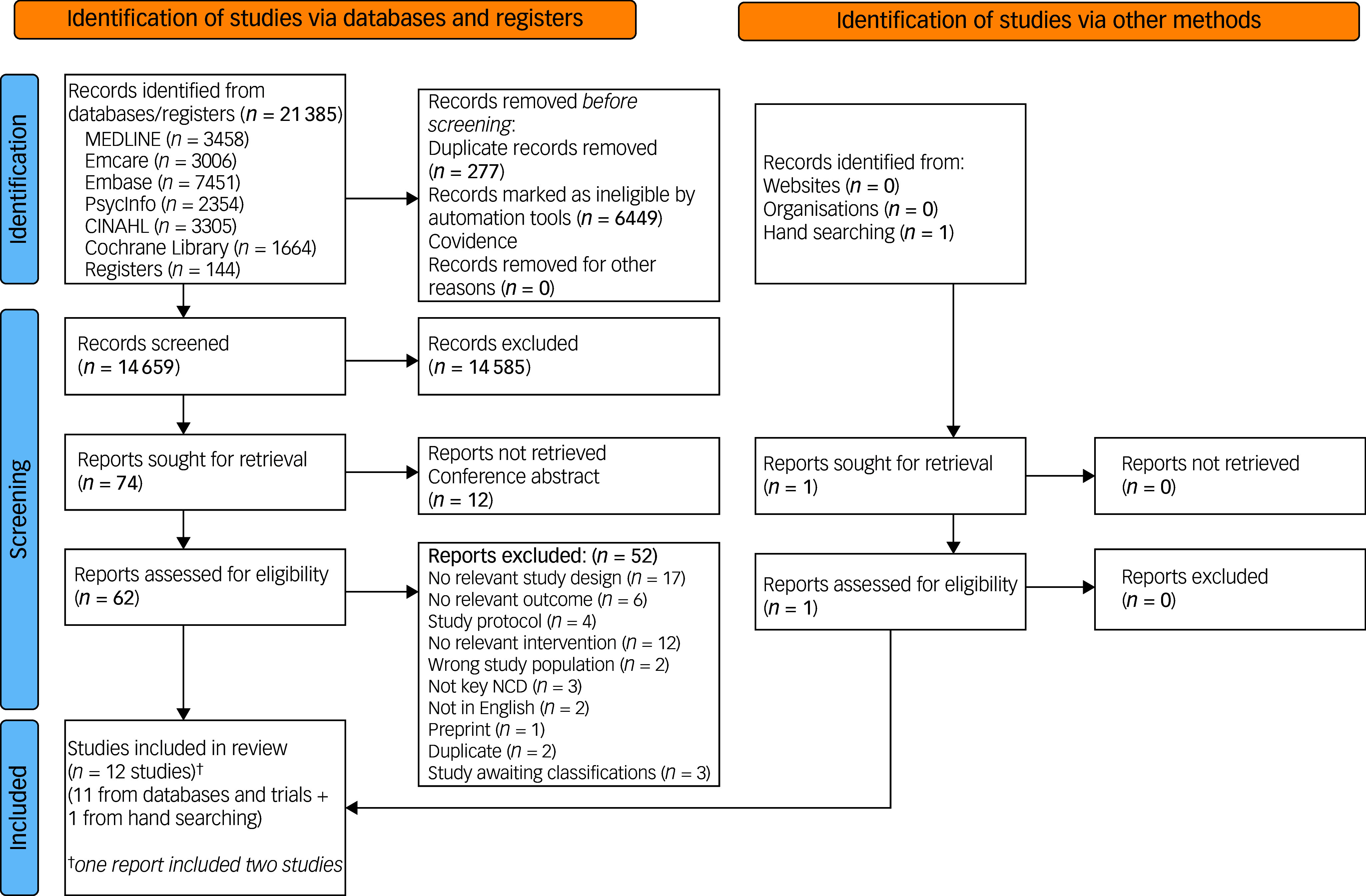
PRISMA flow chart for different stages of the systematic review. NCD, non-communicable disease.

The flow of papers through the review is shown in Fig. 1. The searches identified 21 386 records. A total of 14 659 records/citations were screened at the title and abstract stage. We reviewed 63 reports for eligibility. A total of 52 reports were excluded during full-text review. The reasons for exclusion included irrelevant study design (17 studies), irrelevant intervention (12 studies), irrelevant outcome (six studies), not written in English (two studies) and other reasons (15 studies). The detailed reasons for the exclusion of each report (paper) are provided in Supplementary material III. In total, 12 studies (in 11 papers/reports) were eligible to be included in the final review.

### Description of studies

#### Included studies

The 12 included studies^25–35^ evaluated the effectiveness of behavioural activation on depression in people with NCDs. Together these studies included 2144 patients. The selected characteristics of the included studies are shown in Table 1.

**Table 1:** Selected characteristics of randomised control trials comparing behavioural activation to comparators

Study/Country	Behavioural activation (***N*****)**	Control (*N*)	Types of RCTs	Depression assessment	Health condition	Recruitment setting	Behavioural activation format	Number of behavioural activation sessions offered	Control	Behavioural activation delivery	Outcome measures	Reported harms
Mitchell et al^25^ USA	48	53	Individually RCT	Clinical interviews	Stroke	Acute care hospital	Individual	9	TAU + antidepressant	Study interventionist nurses	HDRS	No
Sun et al^26^ China	35	35	Individually RCT	Rating scale	Stroke	Hospital	Individual	6	TAU	Psychologists	HDRS	No
Thomas et al^27^ UK	54	51	Individually RCT	Rating scale	Stroke	Hospital/community	Individual	20	TAU	Assistant psychologists	SADQ-21	No
Thomas et al^28^ UK	26	23	Individually RCT	Rating scale	Stroke	Hospital, community services, voluntary groups	Individual	15	TAU	Assistant psychologists	PHQ-9	Yes
Kirkness et al^29^ USA	72	28	Individually RCT	Rating scale	Stroke	Community hospital	Individual	6	TAU	Nurse practitioners	HDRS	No
Hopko et al^30^ USA	42	38	Individually RCT	Clinical interviews	Breast cancer	Cancer institute clinics	Individual	8	Problem-solving therapy (PST)	Doctoral students^a^	HDRS	No
Schneider et al^31^ USA	15	14	Individually RCT	Clinical interviews	Type 2 diabetes	Hospital/clinic/community	Group	38	TAU^b^	Group leader^c^	BDI	No
Naik et al^32^ USA	136	89	Individually RCT	Rating scale	Uncontrolled diabetes	Via database, contacted by phone	Individual	9	TAU^b^	24 trained health professionals^d^	PHQ-9	No
Littlewood et al^33^ UK	24	20	Individually RCT	Rating scale	Mixed conditions^e^	Community pharmacy	Individual	Flexible number	TAU	Pharmacy staff	PHQ-9	No
Richards et al^34^ UK	15	14	Cluster RCT	Rating scale	Acute coronary syndrome	Clinical	Individual	8	TAU	Cardiac nurses	BDI	No
Araya et al^35^ Peru	217	215	Individually RCTs	Rating scale	Comorbid hypertension and/or diabetes	Clinical	Guided self-help	18	TAU^b^	Via provided smartphone^f^	PHQ-9	Yes
Araya et al^35^ Brazil	440	440	Cluster RCT	Rating scale	Comorbid hypertension and/or diabetes	Clinical	Guided self-help	18	TAU^b^	Via provided smartphone^f^	PHQ-9	Yes

RCT, randomised controlled trial; TAU, treatment as usual; HDRS, Hamilton Rating Depression Scale; SADQ-21, Stroke Aphasic Depression Questionnaire-21; PHQ-9, Patient Health Questionnaire-9; BDI, Beck Depression Inventory – II.

a .Six doctoral students in clinical psychology trained in behavioural activation and PST.

b .Enhance usual care.

c .Group leader with certification in Pilates/yoga.

d .Twenty-four trained health professionals (psychologists, nurses, pharmacists, social workers).

e .Includes diabetes mellitus, stroke, cancer, respiratory conditions, cardiovascular conditions and others.

f .Automated app sessions based on behavioural activation principles.

#### Setting

Of the 12 studies, five were conducted in the USA, four in the UK and one each in China, Peru and Brazil. The participants were recruited from hospital settings (including community services and clinics) in ten of the studies, one from a community pharmacy and one via a database (contacted by phone).

#### Participants/population

The studies included adults aged 18 years or older with NCDs – stroke (five studies), breast cancer (one study), diabetes (two studies), coronary syndrome (one study), comorbid hypertension or diabetes (two studies) and mixed conditions (one study). Unlike the other studies, Hopko et al^30^ specifically focused on women diagnosed with breast cancer and depression. Women across various stages of breast cancer were included, from Stage 0 to Stage 4, with the majority in the early stages (Stages 0–2). The study employed behavioural activation and problem-solving therapy (PST) to treat depression. The study also assessed adherence and found low attrition rates, indicating good engagement.

#### Intervention

Of the 12 studies, the majority (nine studies) focused on behavioural activation in individual psychotherapy, while only one focused on behavioural activation in groups settings,^31^ and two studies, reported in a single paper, focused on guided self-help.^35^ Most studies (eight studies) delivered behavioural activation in person, while the remaining studies used digital formats (mobile apps) and guided self-help.

In all studies, behavioural activation consisted of 6–38 sessions, with one study^33^ having a flexible number of sessions (delivered where feasible on weekly basis for up to 4 months). Behavioural activation was delivered by nurses,^25,29,34^ psychologists,^26^ assistant psychologists,^27,28^ doctoral students,^30^ group leaders,^31^ pharmacy staff,^33^ mixed (various health professionals)^32^ and automated smartphone apps supported by phone calls.^35^

#### Comparator

In the majority of the studies (10 out of 12 studies), the comparators involved treatment as usual. One study^30^ employed PST as the comparator, while another study^25^ utilised usual care plus antidepressant medication as the comparator. Across all studies, the control conditions did not include any other specific psychological interventions, such as CBT.

#### Outcomes

In four out of the 12 studies, the Hamilton Depression Rating Scale (HDRS) was the primary measure for depression. Five studies utilised the PHQ-9, two studies^31,34^ used the Beck Depression Inventory (BDI) and one study^27^ employed the Stroke Aphasic Depression Questionnaire.

### Risk of bias assessment in the included studies

Of the 12 studies included in the review, six (50%) were rated as having a low risk of bias. Conversely, five studies were assessed as having a high risk of bias, while one study was rated to have some concerns based on the overall risk of bias assessment.

**Fig. 2:**
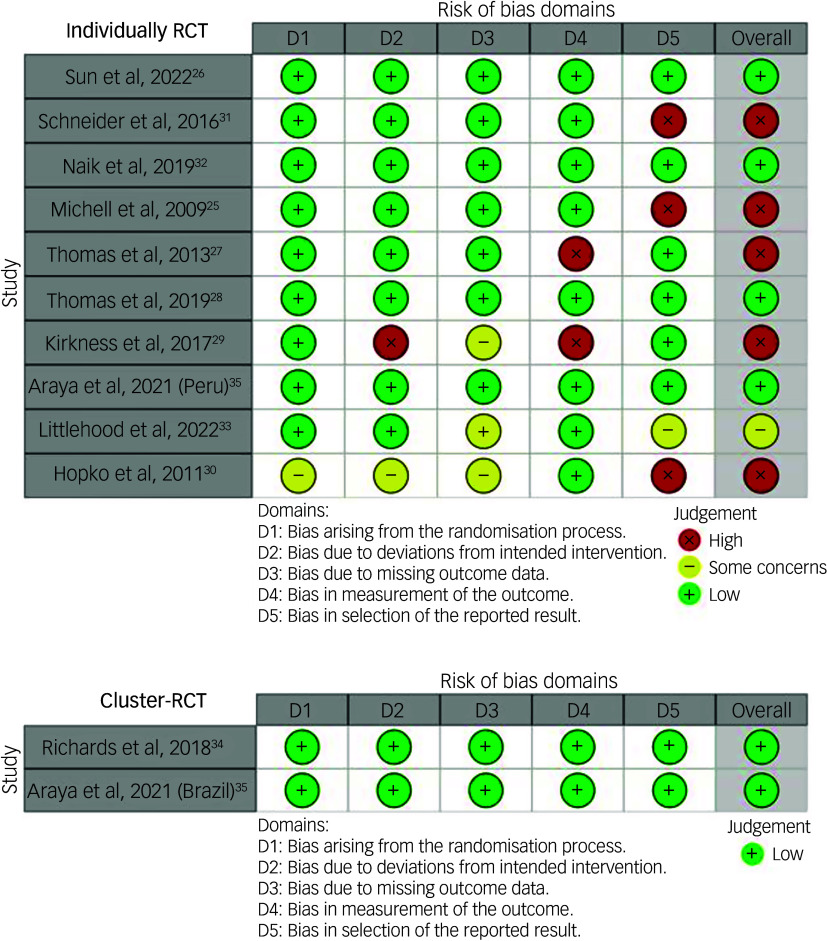
Risk of bias summary: review authors’ judgements about each risk of bias item for each included study. RCT, randomised controlled trial.

Figure 2 shows the risk of bias assessment for five domains across the 12 included studies. For the domain of bias in the selection of the reported results, the Michell et al^25^ study was rated as having ‘high risk’. Regarding bias arising from the randomisation process, the majority of the studies were rated as having ‘low risk’ because they provided sufficient details about the randomisation methods used.

**Fig. 3:**
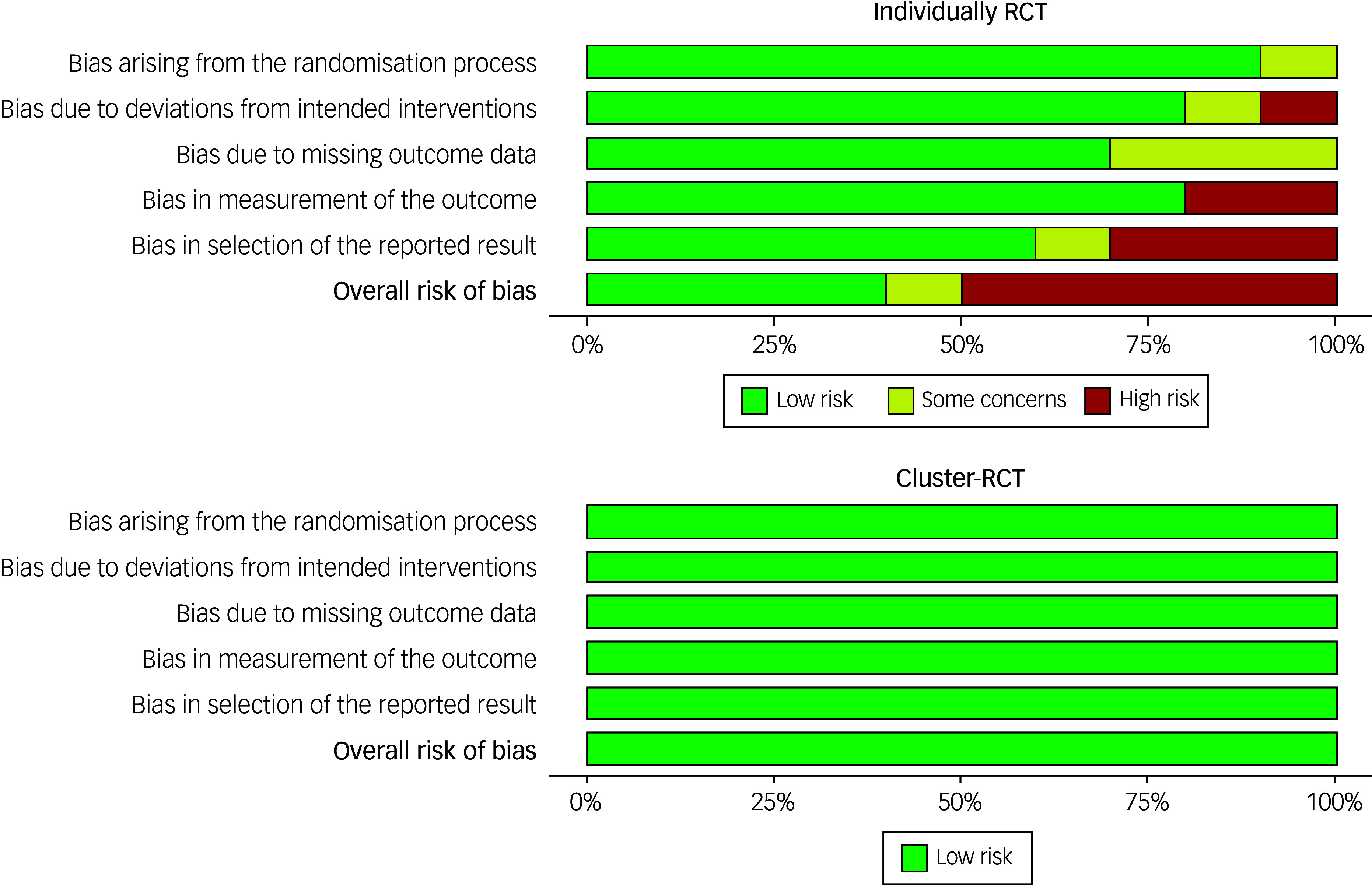
Risk of bias graph: review authors’ judgements about each risk of bias item presented as percentages across all included studies. RCT, randomised controlled trial.

Figure 3 shows that for individually RCTs across all bias domains, more than 50% of the studies were rated as ‘high risk’, and for cluster RCTs, all the studies were rated as ‘low risk’.

### Effect of behavioural activation on depression in people with NCDs

**Fig. 4:**
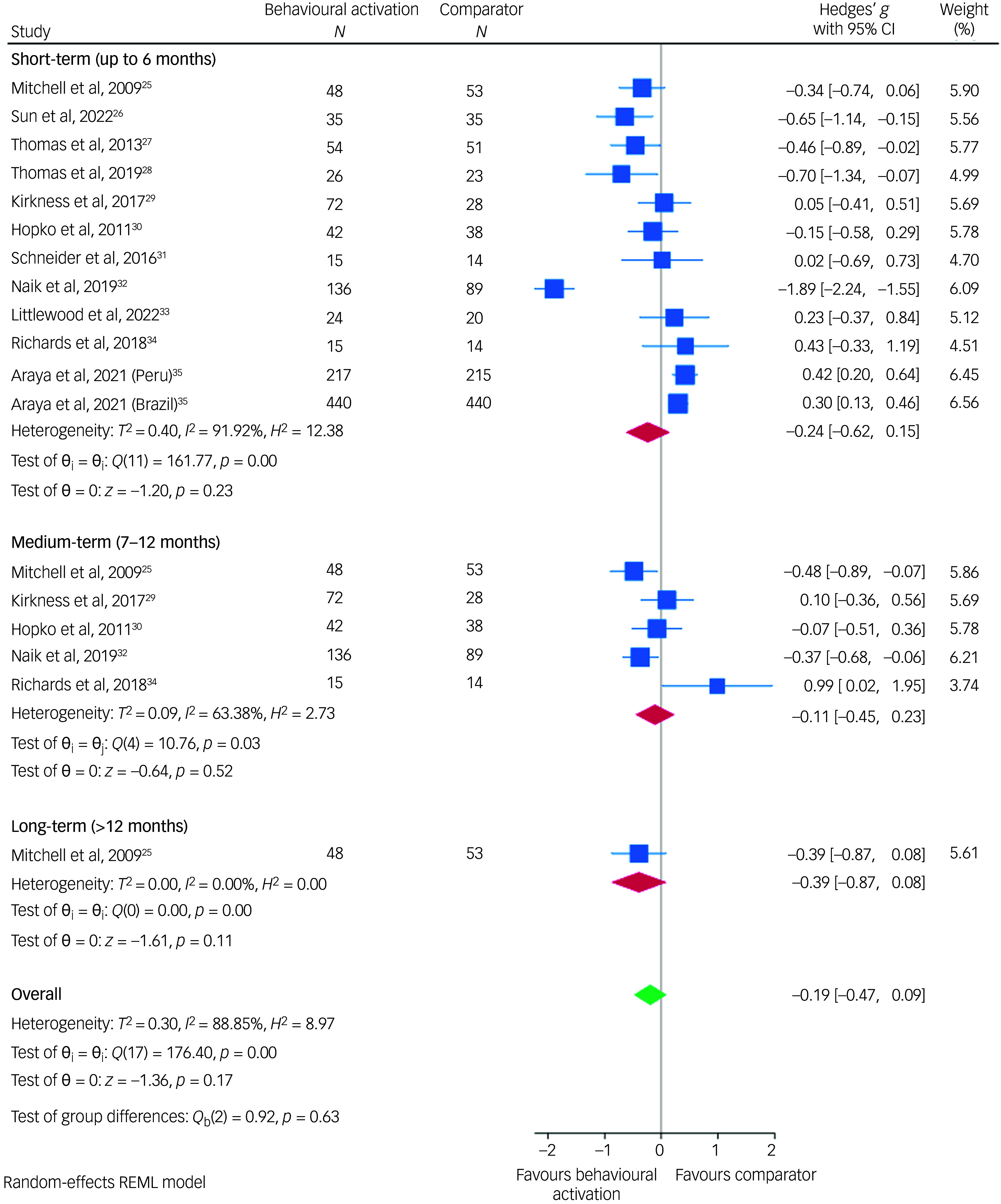
Meta-analysis showing the association between behavioural activation and depression in people with non-communicable diseases for each follow-up. REML, restricted maximum likelihood.

The results of the meta-analysis according to the different follow-up time points are presented in Fig. 4. For short-term follow-ups (up to 6 months), the meta-analysis showed behavioural activation had little effect on depression symptom improvement in people with NCDs (Hedges’ *g* = −0.24; 95% CI, −0.62 to 0.15) compared to control groups, with high heterogeneity (*I* ^2^ = 91.91%).

**Fig. 5:**
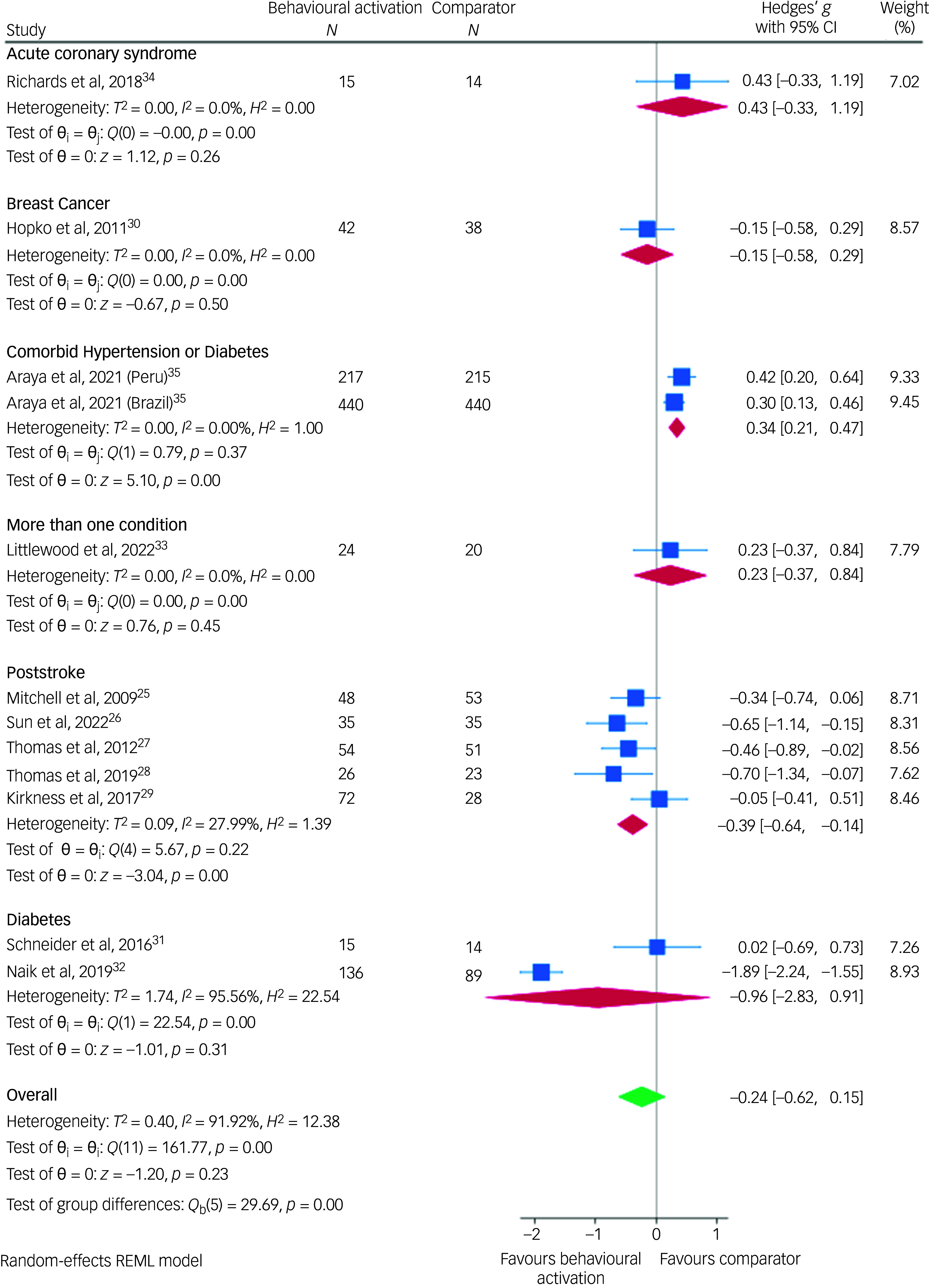
Meta-analysis showing the association between behavioural activation and depression by non-communicable disease type for short-term follow-ups (up to 6 months). REML, restricted maximum likelihood.

Among the 12 studies, five examined medium-term treatment efficacy (7–12 months) of behavioural activation compared to control groups, while only one study (Mitchell et al)^25^ examined the long-term treatment efficacy (>12 months). For medium-term efficacy, behavioural activation showed a Hedges’ *g* of −0.11 (95% CI: −0.45 to 0.23) compared with control groups. The meta-analysis results, conducted on similar studies by NCD type for short-term follow-ups (up to 6 months), yielded consistent findings with high heterogeneity (Fig. 5). However, post-stroke depression differed, showing low heterogeneity, which has been previously reported in a recent meta-analysis.^36^

### Results of the subgroup analyses

The results of the subgroup analyses for studies reporting on short-term follow-ups are reported in Table 2. We found significant difference between studies with participants having a single condition (stroke), studies with a guided self-help format of the intervention and studies with a cluster RCT design. Studies with participants having post-stroke depression had an effect size (Hedges’ *g*) of −0.39 (95% CI: −0.64 to −0.14, *I* ^2^ = 27.99%, *P* = 0.002), favouring behavioural activation over comparators for reducing depression symptoms. The guided self-help format of the intervention showed a Hedges’ *g* of 0.34 (95% CI: 0.21–0.47, *I* ^2^ = 0.00, *P* = 0.000), while cluster RCT design demonstrated a Hedges’ *g* of 0.30 (95% CI: 0.14–0.46, *I* ^2^ = 0.00, *P* = 0.000). The other subgroup analyses examined the type of control group, diagnosis (mood disorder versus scoring above a cut-off), risk of bias, types of intervention, number of behavioural activation sessions provided and types of depression measure scales. None of these analyses pointed at significant differences between subgroups.

**Table 2:** Subgroup analyses for studies reporting on short-term follow-ups

	No. of studies	Hedges’ *g*	[95% CI]	*I* ^2^	*P*-value
Control group					
Usual care	11	−0.24	[−0.67; 0.18]	92.75	0.257
Problem solving	1	−0.15	[−0.58; 0.29]	.	0.502
Diagnosis					
Clinical interview	3	−0.21	[−0.48; 0.06]	0.0	0.126
Rating scale	9	−0.26	[−0.77; 0.25]	94.51	0.323
Risk of bias					
Low	6	−0.35	[−1.10; 0.40]	96.86	0.356
Other	6	−0.16	[−0.37; 0.04]	5.69	0.112
Condition/comorbidity					
PSD	5	−0.39	[−0.64; −0.14]	27.99	0.002
Other	7	−0.11	[−0.74; 0.53]	95.79	0.745
Type of intervention					
Pure behavioural activation	5	−0.10	[−0.55; 0.35]	90.19	0.667
Mixed	7	−0.31	[−0.91; 0.28]	90.27	0.667
Depression measure					
HDRS	4	−0.26	[−0.53; 0.00]	30.41	0.053
PHQ-9	5	−0.32	[−1.20; 0.55]	97.62	0.467
Other	3	−0.08	[−0.60; 0.45]	53.67	0.777
Format of intervention					
Group	1	0.02	[−0.69; 0.73]	.	0.961
Guided self-help	2	0.34	[0.21; 0.47]	0.00	0.000
Individual	9	−0.41	[−0.87; 0.05]	87.76	0.078
Study design					
Cluster RCT	2	0.30	[0.14; 0.46]	0.00	0.000
Individually RCT	10	−0.35	[−0.78; 0.07]	90.05	0.111
Number of behavioural activation sessions provided					
≤6	3	−0.13	[−0.66; 0.39]	67.70	0.618
≥7	9	−0.27	[−0.76; 0.22]	94.23	0.280

HDRS, Hamilton Rating Depression Scale; PHQ-9, Patient Health Questionnaire-9; PSD, post-stroke depression; RCT, randomised controlled trial.

The investigations for possible sources of heterogeneity, associated with the pooled estimate of the association between behavioural activation and depression in people with NCDs, did not indicate that the majority of these subgroup analysis-based variables are important sources of heterogeneity (i.e. the heterogeneity remains high).

### Adverse events and harms

Out of the 12 included studies, only three trials (Thomas et al^28^ and Araya et al^35^ (two trials reported in one paper)) provided data on adverse events occurring during the trial implementation. Thomas et al^28^ reported on both serious adverse events, defined as those requiring admission to hospital or emergent care, as well as general adverse events. They documented three serious adverse events, namely hospital admissions for a suicide attempt, heart attack and hernia surgery, experienced by three separate participants. Importantly, none of these major adverse events were judged to be related to the study intervention. Regarding minor adverse events, a total of 13 events were reported in 10 participants overall.^28^ These included suicidal ideation, worsening health status, falls and new medical conditions emerging during the study period. When examined by study group, five adverse events occurred in four participants assigned to the intervention arm, while eight events were documented in six control arm participants.

In Araya et al (Peru),^35^ 2.8% of participants in the behavioural activation group experienced worsening of depression compared to 5.1% in the enhanced usual care group. Increased suicidal ideation affected 3.7% of the behavioural activation group and 7.4% of the enhanced usual care group. Unexpected physical health-related events occurred in 4.6% of the behavioural activation group and 4.7% of the enhanced usual care group. Araya et al (Peru) reported that all adverse events involving worsening depression or increased suicidality were managed according to the safety protocol to ensure participant safety.

In Araya et al (Brazil),^35^ adverse events in the form of worsening of depression were experienced by 13.4% of participants in the behavioural activation group and 15.0% in the enhanced usual care group. Increased suicidal ideation occurred in 7.1% of the behavioural activation group and 6.6% of the enhanced usual care group. Unexpected events related to participants’ physical health issues, such as hospital admissions, surgery, cardiac arrest and death caused by physical diseases, were seen in 1.6% of the behavioural activation group and 0.7% of the enhanced usual care group.

## Discussion

### Key findings and interpretation

This systematic review and meta-analysis examined the effectiveness of behavioural activation versus any comparator in improving depression symptoms in people with NCDs at multiple time points post-treatment. For short-term follow-ups (up to 6 months), our meta-analysis found a Hedges’ *g* of −0.24 (95% CI: −0.62 to 0.15) with high heterogeneity (*I* ^2^ = 91.92%) for behavioural activation compared to control groups. This suggests that behavioural activation did not show significant benefits over controls in reducing depression symptoms in the short term. Moreover, the high heterogeneity indicates that the effectiveness of behavioural activation may vary considerably across studies. For medium-term efficacy (6–12 months), the meta-analysis found a Hedges’ *g* of −0.11 (95% CI: −0.45 to 0.23) for behavioural activation versus control groups and the heterogeneity (*I* ^2^ = 63.38%) was moderately high, suggesting variability in study results. For long-term treatment efficacy (>12 months), as only one study was available, no meta-analytic estimate could be provided. Moreover, of the 12 included studies, only three trials provided data on adverse events occurring during the trial. Therefore, the evidence from this review is not sufficient to draw conclusions about the efficacy and safety of behavioural activation over comparators in improving depression symptoms in people with NCDs.

### Comparison with previous findings

The findings of this systematic review and meta-analysis are consistent with the broader literature on the treatment of depression in people with chronic physical health conditions. A 2021 Cochrane review by Tully et al^37^ examined the effectiveness of psychological and pharmacological interventions for depression in patients with coronary artery disease. Consistent with the current review, these researchers found limited evidence to support the superiority of any specific psychological intervention, including behavioural approaches, over usual care or other comparators. Moreover, a 2020 review by Uphoff et al,^18^ which specifically focused on the effect of behavioural activation for clinically diagnosed depression in adults with NCDs, reported consistent findings with this current review – insufficient evidence to confirm the effect of behavioural activation for depression in adults with NCDs. However, a key difference is that the current review included studies that enrolled participants based on both clinician-rated diagnoses of depression as well as those using self-reported depression scales, while the Uphoff et al review focused only on studies with clinically diagnosed depression. By including a broader range of depression assessment methods, the current review provides a more comprehensive synthesis of the available evidence on the effect of behavioural activation on depression, including subthreshold depression or depression symptoms without a formal diagnosis in the context of NCDs. The inclusion of depression assessed based on clinical interviews (clinician-rated) and rating scales in the current review allows for a better understanding of the real-world applicability of behavioural activation interventions for managing depressive symptoms in people living with NCDs.

In contrast, the findings of the current review are inconsistent with the findings of previous systematic reviews and meta-analyses on the effectiveness of behavioural activation on depression in adults without comorbidity. For example, two meta-analyses^24,38^ have found that behavioural activation is an effective treatment for depression, with effect sizes in the moderate to large range. The relatively small effects observed in this review suggest that the effectiveness of behavioural activation may be attenuated when delivered to people with comorbid NCDs. Thus, the findings of this review and the 2020 review by Uphoff et al^18^ suggest the need for further research to better understand the role of behavioural activation in the treatment of depression among people with NCDs.

Despite the limited evidence for the effectiveness of behavioural activation in improving depression symptoms among people with NCDs, the current review provides an important contribution to the existing literature. Although the findings do not conclusively support the use of behavioural activation for depression in this population, the review identifies important knowledge gaps in the existing evidence. This information can help guide future research efforts and inform the development of more tailored approaches to address the complex interplay between depression and chronic physical conditions.

Moreover, in this review we found limited data on adverse events from behavioural activation interventions, with three out of 12 included studies reporting adverse events/harms. The adverse events reported included serious events such as admissions to hospital, worsening depression and increased suicidality. However, the paucity of data makes it difficult to fully evaluate the risks. In contrast, a 2023 review^39^ on CBT for post-stroke depression found no studies documenting adverse effects. However, another study^40^ found that CBT therapists reported 372 unwanted events across 98 patients, with negative well-being/distress and symptom worsening being common. These findings highlight the importance of thorough monitoring and reporting potential adverse effects of psychotherapeutic interventions such as behavioural activation, similar to how such effects are documented for pharmacological treatments. Comprehensive data on adverse events is needed to assess the risk–benefit profile of these interventions compared to other therapeutic approaches.

### Limitations of the review

This review has several limitations that should be considered when interpreting the findings. First, the included studies demonstrated high heterogeneity, particularly in the short-term follow-up analysis, which limits the ability to draw conclusions about the overall effectiveness of behavioural activation for depression in people with NCDs. The sources of this heterogeneity were explored through subgroup analyses, but many potential moderating factors could not be reliably examined because of the small number of included studies. Moreover, the relatively small number of eligible studies restricted the ability to conduct comprehensive subgroup analyses and explore the effectiveness of behavioural activation for depression across different types of NCDs. Although the review was able to identify some potential differences in the effect of behavioural activation based on the type of NCD (e.g. stroke versus other conditions), the limited data precluded a more detailed examination. If a larger body of research becomes available in the future, it would be valuable to conduct separate meta-analyses for each major NCD category (e.g. cardiovascular diseases, diabetes, cancer, chronic respiratory diseases) to better understand the differential effects of behavioural activation on depression in these populations. This approach would provide insights into the contexts in which behavioural activation may be most effective for managing comorbid depression. Second, a significant number of studies exhibited poor quality, indicating a potential high risk of bias in the outcomes. This introduces uncertainty about the internal validity of the evidence base. Finally, the current review lacks long-term follow-up data. Understanding the sustainability of behavioural activation’s effects on depression in people with NCDs is crucial for evaluating its clinical utility and potential for integration into comprehensive NCD management strategies.

### Future research

Future systematic reviews and meta-analyses examining the effect of behavioural activation on depression in people with NCDs should make efforts to include larger, higher-quality RCTs. This approach will allow for more definitive conclusions about the effectiveness of behavioural activation for treating depression in people with NCDs. These studies should explore potential moderating factors that may influence the success of behavioural activation, such as the type of NCD, severity of depression and mode of behavioural activation delivery. Future studies should also consider that the training provided to those administering behavioural activation interventions must be comprehensive and appropriate. Similarly, individuals tasked with delivering behavioural activation should receive adequate and ongoing support to ensure proper implementation. Understanding the factors that predict or hinder the effectiveness of behavioural activation can inform the tailoring of interventions to individual patient needs. The methodological quality and risk of bias of included studies needs careful assessment. Moreover, beyond evaluating the overall effectiveness of behavioural activation, it is also important to investigate the mechanisms by which behavioural activation may improve depression in the context of NCDs. Examining the active components of the intervention and how they interact with the underlying pathophysiology of comorbid physical and mental health conditions could lead to the development of more targeted and effective interventions. Moreover, future research needs to investigate the patient-specific characteristics and treatment components that might influence responses to behavioural activation within diverse NCD populations, particularly given the potential long-term benefits on quality of life and relapse prevention, which are not captured in the current review.

Given that the prevalence of depression and NCDs such as diabetes, cancer, heart disease and chronic respiratory illnesses is common in both high-income and low- to middle-income countries, future studies may wish to include data from low- and middle-income countries. This is important because behavioural activation is emerging as a treatment option that may require fewer resources and less specialised training compared to the established psychological therapies such as CBT. Investigating the evidence from the perspective of low- and middle-income countries, as well as rural and remote areas, could shed light on whether behavioural activation could serve as a viable, acceptable and feasible alternative for treating depression among people with NCDs.

In conclusion, the evidence from this review is not sufficient to draw clear conclusions about the effectiveness and safety of behavioural activation over comparators in improving depression symptoms in people with NCDs. Before considering behavioural activation as an alternative treatment to other depression treatments, more research is needed to confirm its effectiveness and acceptability in the context of non-communicable health conditions. Behavioural activation has been suggested as a simpler treatment option compared to other psychological treatments, potentially making it easier to implement more widely. There is a potential to integrate behavioural activation into various healthcare settings and clinics attended by those with NCDs. However, further high-quality research, including large-scale RCTs with longer follow-up periods, is essential to fully understand both the advantages and limitations of behavioural activation in this specific patient population.

## Supporting information

Yisma et al. supplementary material 1 Yisma et al. supplementary material

Yisma et al. supplementary material 2 Yisma et al. supplementary material

Yisma et al. supplementary material 3 Yisma et al. supplementary material

## Data Availability

The data that support the findings of this study are available on request from the corresponding author.

## Supplementary material

The supplementary material for this article can be found at https://doi.org/10.1192/bjo.2024.870
