# Supplementary material for: Efficacy and safety of behavioural activation on depression in people with co-occurring non-communicable diseases: systematic review and meta-analysis
Source: BJPsych Open. 2025 Mar 24;11(2):e70. doi: 10.1192/bjo.2024.870 (PMC12021889; doi:10.1192/bjo.2024.870)
Supplement: Yisma et al. supplementary material 1 — Yisma et al. supplementary material [file S2056472424008706sup001.docx]

**Supplementary material III**: **List of excluded studies**

|  | **Study** | **Reason** |
| --- | --- | --- |
| 1 | [Ishak 2021](https://doi.org/10.1371/journal.pone.0244453) | Study protocol |
| 2 | [Brown 1993](https://journals.lww.com/jcrjournal/abstract/1993/05000/behavior_therapy_of_psychological_distress_in.9.aspx) | No relevant intervention |
| 3 | [Armento 2009](https://doi.org/10.1177/1534650108327474) | No relevant study design |
| 4 | [Kaltman 2016](https://doi.org/10.1177/0145721715617536) | No relevant study design |
| 5 | [Hopko 2008](https://doi.org/10.1037/h0100862) | No relevant study design |
| 6 | [Hirayama 2022](https://doi.org/10.1101/2022.10.06.22280763) | Preprint |
| 7 | [Hopko 2005](https://doi.org/10.1037/0033-3204.42.2.236) | No relevant study design |
| 8 | [Ayudhaya 2022](https://bmcpsychiatry.biomedcentral.com/articles/10.1186/s12888-022-03962-8) | Not key NCD |
| 9 | [Fernandez-Rodriguez 2021](https://doi.org/10.1177/0145445520916441) | Study awaiting classification |
| 10 | [NCT01572389](https://clinicaltrials.gov/study/NCT01572389) | Study protocol |
| 11 | [Ell 2014](https://doi.org/10.1016/j.psym.2014.03.007) | No relevant intervention |
| 12 | [Cannity 2017](https://link.springer.com/article/10.1007/s10879-017-9359-6) | No relevant study design |
| 13 | [Fernandez 2011](https://revistas.um.es/analesps/article/view/122851) | Not in English |
| 14 | [Ryba 2014](https://doi.org/10.1037/a0035363) | No relevant study design |
| 15 | [HopkoDerek 2015](https://doi.org/10.1037/a0037704) | No relevant study design |
| 16 | [Chew-Graham 2022](https://pilotfeasibilitystudies.biomedcentral.com/articles/10.1186/s40814-022-00992-7) | No relevant study design |
| 17 | [Hopko 2009](https://doi.org/10.1016/j.beth.2008.09.001) | No relevant study design |
| 18 | [Li 2023](https://doi.org/10.1002/cam4.5030) | Wrong study population |
| 19 | [Humphreys 2015](https://doi.org/10.1177/0269215514537656) | No relevant outcome |
| 20 | [Hopko 2013](https://doi.org/10.1177/0145445513501512) | No relevant outcome |
| 21 | [Akechi 2023](https://doi.org/10.1200/JCO.22.0069) | No relevant outcome |
| 22 | [Li 2022](https://doi.org/10.1093/eurjcn/zvac104) | Not key NCD |
| 23 | [Fernandez-Rodriguez 2019](https://doi.org/10.1177/0145445517746915) | Study awaiting classification |
| 24 | [Ell 2011](https://doi.org/10.1016/j.genhosppsych.2011.05.018) | No relevant intervention |
| 25 | [Ell 2012](https://doi.org/10.1016/j.psym.2011.12.009) | No relevant study design |
| 26 | [Gonzalez-Fernandez 2018](https://doi.org/10.7334/psicothema2017.396) | Study awaiting classification |
| 27 | [Thomas 2013](https://journals.sagepub.com/doi/10.1177/0269215513489579) | No relevant outcome |
| 28 | [Fann 2009](https://link.springer.com/article/10.1007/s11606-009-0999-4) | No relevant intervention |
| 29 | [Hopko 2016](https://www.tandfonline.com/doi/full/10.1111/cp.12083) | No relevant outcome |
| 30 | [Pentecost 2015](https://trialsjournal.biomedcentral.com/articles/10.1186/s13063-015-0881-0) | Wrong study population |
| 31 | [Coventry 2015](https://www.bmj.com/content/350/bmj.h638) | No relevant intervention |
| 32 | [Morales-Cruz 2017](https://psycnet.apa.org/record/2017-15254-007) | Not in English |
| 33 | [Egede 2017](https://journals.sagepub.com/doi/10.1177/1357633X17730419) | No relevant outcome |
| 34 | [CTRI/2020/05/025048](https://trialsearch.who.int/Trial2.aspx?TrialID=CTRI/2020/05/025048) | Study protocol |
| 35 | [Gathright 2022](https://journals.plos.org/plosone/article?id=10.1371/journal.pone.0261490) | No relevant study design |
| 36 | [Yusufov 2023](https://regroup-production.s3.amazonaws.com/documents/ReviewReference/1117725513/Yusufov%202023%20Psycho-Oncology%20-%202023%20-%20%20-%20Symposia%20%20%20Podium%20Abstracts.pdf?response-content-type=application%2Fpdf&X-Amz-Algorithm=AWS4-HMAC-SHA256&X-Amz-Credential=AKIAYSFKCAWYQ4D5IUHG%2F20241104%2Fus-east-1%2Fs3%2Faws4_request&X-Amz-Date=20241104T235720Z&X-Amz-Expires=604800&X-Amz-SignedHeaders=host&X-Amz-Signature=c4753a0258d7b2d4a8eb5acda0b67beeea09a01c1bf103d5feac024b791729f8) | No relevant intervention |
| 37 | [Fann 2023](https://www.centerwatch.com/clinical-trials/listings/NCT05012124/using-technology-to-optimize-collaborative-care-management-of-depression-in-urban-and-rural-cancer-centers-scope) | Currently Recruiting |
| 38 | [Llave 2024](https://link.springer.com/article/10.1007/s12529-024-10264-8) | Wrong study design |
| 39 | [Greer 2024](https://ascopubs.org/doi/10.1200/JCO.24.00048) | Wrong intervention |
| 40 | [Sayampurna 2023](https://www.tandfonline.com/doi/full/10.1080/02687038.2023.2244166) | Wrong study design |
| 41 | Li 2023 | Duplicate |
| 42 | [Lahousse 2024](https://link.springer.com/article/10.1007/s11764-022-01270-4) | Wrong study design |
| 43 | Hirayama 2023 | Duplicate |
| 44 | [Baptist 2024](https://www.tandfonline.com/doi/full/10.1080/02770903.2024.2380517) | Wrong study design |
| 45 | [Rogerson 2024](https://academic.oup.com/eurjcn/article/23/1/42/7093304) | Wrong intervention |
| 46 | [Saha 2024](https://www.sciencedirect.com/science/article/pii/S1056872723002647?via%3Dihub) | Wrong intervention |
| 47 | [Walsh 2024](https://link.springer.com/article/10.1007/s10549-023-07228-z) | Wrong intervention |
| 48 | [Hirayama 2023](https://www.liebertpub.com/doi/10.1089/pmr.2023.0020) | Wrong study design |
| 49 | [Granger 2024](https://www.tandfonline.com/doi/full/10.1080/09638288.2022.2094478) | Not key NCD |
| 50 | [Becerra-Galvez 2023](https://www.gamo-smeo.com/frame_esp.php?id=340) | Wrong study design |
| 51 | [Jun 2024](https://doi.org/10.1016/j.hrtlng.2023.11.00) | Wrong intervention |
| 52 | [Li 2023](https://academic.oup.com/eurjcn/article/22/6/655/6832142) | Wrong intervention |
