# Supplementary material for: Efficacy and safety of behavioural activation on depression in people with co-occurring non-communicable diseases: systematic review and meta-analysis
Source: BJPsych Open. 2025 Mar 24;11(2):e70. doi: 10.1192/bjo.2024.870 (PMC12021889; doi:10.1192/bjo.2024.870)
Supplement: Yisma et al. supplementary material 2 — Yisma et al. supplementary material [file S2056472424008706sup002.docx]

**Supplementary material II**

**Search Strategy for “Efficacy and safety of behavioural activation on depression in people with co-occurring non-communicable diseases: a systematic review and meta-analysis”**

**Table S1. MEDLINE**

**Database:**
Ovid MEDLINE(R) ALL <1946 to March 28, 2023>

| **#** | **Query** | **Results from 30 Mar 2023** |
| --- | --- | --- |
| 1 | Noncommunicable Diseases/ | 2,936 |
| 2 | ((non communicable or noncommunicable or non infectious or noninfectious) adj3 disease*).ti,ab,kf. | 19,074 |
| 3 | Chronic Disease/ | 279,981 |
| 4 | (chronic* adj3 (condition or conditions or disease* or ill*)).ti,ab,kf. | 412,262 |
| 5 | exp Pulmonary Disease, Chronic Obstructive/ | 66,004 |
| 6 | Lung Diseases, Obstructive/ | 18,276 |
| 7 | exp Asthma/ | 141,252 |
| 8 | Respiratory Hypersensitivity/ | 9,956 |
| 9 | Hypertension, Pulmonary/ | 38,492 |
| 10 | ((long term or longterm or chronic*) adj5 (bronchitis or respirat*)).ti,ab,kf. | 30,963 |
| 11 | emphysema*.ti,ab,kf. | 30,678 |
| 12 | asthma*.ti,ab,kf. | 179,604 |
| 13 | (obstruct* adj3 (pulmonary or lung* or airway* or airflow* or bronch* or respirat*)).ti,ab,kf. | 103,508 |
| 14 | ((hyper responsiveness or hyper-responsiveness or allergi* or hypersensitiv* or hyperreactiv* or insufficiency) adj5 (airway or respirat* or bronchial* or lung*)).ti,ab,kf. | 30,681 |
| 15 | ((longterm or long term or Chronic* or occupational or recur*) adj2 lung* adj5 (condition* or disease* or symptom* or problem* or failure* or ill*)).ti,ab,kf. | 17,835 |
| 16 | (respirat* adj2 (condition* or disease* or symptom* or problem* or failure or ill*)).ti,ab,kf. | 124,013 |
| 17 | pulmonary hypertension.ti,ab,kf. | 43,207 |
| 18 | (COPD or COAD or COBD or AECB or AECOPD).ti,ab,kf. | 58,974 |
| 19 | 1 or 2 or 3 or 4 or 5 or 6 or 7 or 8 or 9 or 10 or 11 or 12 or 13 or 14 or 15 or 16 or 17 or 18 | 1,051,017 |
| 20 | exp Diabetes Mellitus/ | 500,982 |
| 21 | Diabetes Mellitus, Type 2/ or Diabetes Mellitus, Type 1/ | 236,770 |
| 22 | Glucose Tolerance Test/ | 36,806 |
| 23 | Glycated Hemoglobin A/ | 42,349 |
| 24 | Blood Glucose/ | 181,921 |
| 25 | diabet*.ti,ab,kf. | 766,030 |
| 26 | (noninsulin*-depend* or non-insulin*-depend* or noninsulin*depend* or non-insulin*depend*).ti,ab,kf. | 12,459 |
| 27 | (fasting glucose or plasma glucose or glucose tolerance test* or ((glycemic or glycaemic) adj2 control*)).ti,ab,kf. | 112,629 |
| 28 | (HbA1c or A1C or A1c or Hb1c or ((glycated or glycosylated) adj (hemoglobin or haemoglobin))).ti,ab,kf. | 69,879 |
| 29 | (NIDDM or T2D or T2DM or T1D or IDDM or MODY or T1DM).ti,ab,kf. | 74,664 |
| 30 | 20 or 21 or 22 or 23 or 24 or 25 or 26 or 27 or 28 or 29 | 937,888 |
| 31 | exp Cardiovascular Diseases/ | 2,693,789 |
| 32 | Blood Pressure/ | 290,588 |
| 33 | (arteriosclero* or atherosclero* or peripheral arter* disease* or blood pressure).ti,ab,kf. | 535,942 |
| 34 | (cardio* or cardia* or cvd or heart* or coronary or angina or ventric* or myocard* or pericard* or ischem* or ischaem* or emboli* or cerebrovasc* or cerebral vascular or arrhythmi* or thrombo* or atrial fibrillat* or infarct* or apoplexy or stroke or strokes or poststroke or hypertensi*).ti,ab,kf. | 3,490,982 |
| 35 | 31 or 32 or 33 or 34 | 4,456,909 |
| 36 | exp Neoplasms/ | 3,812,146 |
| 37 | (cancer* or neoplas* or tumo* or carcinoma* or hodgkin* or nonhodgkin* or non hodgkin* or adenocarcinoma* or leukemia* or leukaemia* or metasta* or malignan* or lymphoma* or sarcoma* or melanoma* or myeloma* or oncolog* or psychooncolog* or glioma*).ti,ab,kf. | 4,423,795 |
| 38 | 36 or 37 | 5,152,461 |
| 39 | 19 or 30 or 35 or 38 | 10,320,019 |
| 40 | Depression/ | 148,470 |
| 41 | Mood Disorders/ | 15,833 |
| 42 | Depressive Disorder/ | 74,948 |
| 43 | Depressive Disorder, Major/ | 37,752 |
| 44 | Depressive Disorder, Treatment-Resistant/ | 2,067 |
| 45 | Dysthymic Disorder/ | 1,172 |
| 46 | Cyclothymic Disorder/ | 780 |
| 47 | (depress* or dysthymi* or cyclothymi* or low mood or mood disorder* or affective disorder*).ti,ab,kf. | 576,324 |
| 48 | 40 or 41 or 42 or 43 or 44 or 45 or 46 or 47 | 621,332 |
| 49 | 39 and 48 | 160,323 |
| 50 | Behavior Therapy/ | 30,174 |
| 51 | Motivational Interviewing/ | 2,518 |
| 52 | behavio* activat*.ti,ab,kf. | 2,573 |
| 53 | (behavio* activat* or BATD).ti,ab,kf. | 2,578 |
| 54 | (behavio* adj3 (reinforce* or re-inforce*)).ti,ab,kf. | 3,543 |
| 55 | (behavio* adj2 (contracting or modif*)).ti,ab,kf. | 11,283 |
| 56 | reinforc*.ti,kf. | 28,496 |
| 57 | ((positive adj1 reinforc*) or (reinforc* adj3 (environment* or experience*))).ti,ab,kf. | 3,568 |
| 58 | ((behavio* adj3 motivat*) or motivational interviewing).ti,ab,kf. | 13,652 |
| 59 | (activit* adj2 schedul*).ti,ab,kf. | 696 |
| 60 | ((pleas* or enjoyable or rewarding) adj3 (activit* or event?)).ti,ab,kf. | 1,492 |
| 61 | ((operant or instrumental) adj (conditioning or learning)).ti,ab,kf. | 3,736 |
| 62 | (positive interaction* or avoida* coping or environmental contingenc* or contingency management).ti,ab,kf. | 6,106 |
| 63 | functional analysis.ti,ab,kf. | 29,921 |
| 64 | behavio*.mp. and (self adj (evaluat* or monitor*)).ti,ab,kf. | 4,820 |
| 65 | (behavio* adj (counsel* or intervention* or train* or treatment* or therap* or psychotherap*)).ti,ab,kf. | 49,429 |
| 66 | (mood adj3 monitor*).ti,ab,kf. | 346 |
| 67 | 50 or 51 or 52 or 53 or 54 or 55 or 56 or 57 or 58 or 59 or 60 or 61 or 62 or 63 or 64 or 65 or 66 | 170,093 |
| 68 | 49 and 67 | 3,098 |
| 69 | limit 68 to dt=20221026-20230330 | 98 |

**Table S2. Emcare**

**Database:**
Ovid Emcare <1995 to 2023 Week 12>

| **#** | **Query** | **Results from 30 Mar 2023** |
| --- | --- | --- |
| 1 | non communicable disease/ | 4,053 |
| 2 | ((non communicable or noncommunicable or non infectious or noninfectious) adj3 disease*).ti,ab,kw. | 9,741 |
| 3 | Chronic Disease/ | 49,667 |
| 4 | (chronic* adj3 (condition or conditions or disease* or ill*)).ti,ab,kw. | 143,158 |
| 5 | chronic obstructive lung disease/ | 27,727 |
| 6 | obstructive lung disease/ | 11 |
| 7 | exp Asthma/ | 42,741 |
| 8 | respiratory tract allergy/ | 1,059 |
| 9 | pulmonary hypertension/ | 12,999 |
| 10 | ((long term or longterm or chronic*) adj5 (bronchitis or respirat*)).ti,ab,kw. | 7,357 |
| 11 | emphysema*.ti,ab,kw. | 6,999 |
| 12 | asthma*.ti,ab,kw. | 47,038 |
| 13 | (obstruct* adj3 (pulmonary or lung* or airway* or airflow* or bronch* or respirat*)).ti,ab,kw. | 31,877 |
| 14 | ((hyper responsiveness or hyper-responsiveness or allergi* or hypersensitiv* or hyperreactiv* or insufficiency) adj5 (airway or respirat* or bronchial* or lung*)).ti,ab,kw. | 5,068 |
| 15 | ((longterm or long term or Chronic* or occupational or recur*) adj2 lung* adj5 (condition* or disease* or symptom* or problem* or failure* or ill*)).ti,ab,kw. | 5,026 |
| 16 | (respirat* adj2 (condition* or disease* or symptom* or problem* or failure or ill*)).ti,ab,kw. | 36,617 |
| 17 | pulmonary hypertension.ti,ab,kw. | 11,508 |
| 18 | (COPD or COAD or COBD or AECB or AECOPD).ti,ab,kw. | 19,774 |
| 19 | or/1-18 | 289,063 |
| 20 | exp Diabetes Mellitus/ | 190,108 |
| 21 | Glucose Tolerance Test/ | 2,701 |
| 22 | hemoglobin A1c/ | 22,167 |
| 23 | glucose blood level/ | 49,172 |
| 24 | diabet*.ti,ab,kw. | 250,712 |
| 25 | (noninsulin*-depend* or non-insulin*-depend* or noninsulin*depend* or non-insulin*depend*).ti,ab,kw. | 2,302 |
| 26 | (fasting glucose or plasma glucose or glucose tolerance test* or ((glycemic or glycaemic) adj2 control*)).ti,ab,kw. | 40,027 |
| 27 | (HbA1c or A1C or A1c or Hb1c or ((glycated or glycosylated) adj (hemoglobin or haemoglobin))).ti,ab,kw. | 30,482 |
| 28 | (NIDDM or T2D or T2DM or T1D or IDDM or MODY or T1DM).ti,ab,kw. | 25,848 |
| 29 | or/20-28 | 318,781 |
| 30 | exp cardiovascular disease/ | 726,706 |
| 31 | blood pressure/ | 38,697 |
| 32 | (arteriosclero* or atherosclero* or peripheral arter* disease* or blood pressure).ti,ab,kw. | 143,689 |
| 33 | (cardio* or cardia* or cvd or heart* or coronary or angina or ventric* or myocard* or pericard* or ischem* or ischaem* or emboli* or cerebrovasc* or cerebral vascular or arrhythmi* or thrombo* or atrial fibrillat* or infarct* or apoplexy or stroke or strokes or poststroke or hypertensi*).ti,ab,kw. | 973,009 |
| 34 | or/30-33 | 1,253,096 |
| 35 | exp neoplasm/ | 577,687 |
| 36 | (cancer* or neoplas* or tumo* or carcinoma* or hodgkin* or nonhodgkin* or non hodgkin* or adenocarcinoma* or leukemia* or leukaemia* or metasta* or malignan* or lymphoma* or sarcoma* or melanoma* or myeloma* or oncolog* or psychooncolog* or glioma*).ti,ab,kw. | 894,217 |
| 37 | 35 or 36 | 1,017,763 |
| 38 | 19 or 29 or 34 or 37 | 2,452,189 |
| 39 | depression/ | 86,620 |
| 40 | mood disorder/ | 13,113 |
| 41 | major depression/ | 19,472 |
| 42 | treatment resistant depression/ | 468 |
| 43 | dysthymia/ | 2,696 |
| 44 | cyclothymia/ | 304 |
| 45 | minor depression/ | 68 |
| 46 | post-stroke depression/ | 166 |
| 47 | subsyndromal depression/ | 88 |
| 48 | (depress* or dysthymi* or cyclothymi* or low mood or mood disorder* or affective disorder*).ti,ab,kw. | 226,093 |
| 49 | or/39-48 | 253,359 |
| 50 | 38 and 49 | 66,990 |
| 51 | Behavior Therapy/ | 13,150 |
| 52 | Motivational Interviewing/ | 2,456 |
| 53 | behavio* activat*.ti,ab,kw. | 1,264 |
| 54 | (behavio* activat* or BATD).ti,ab,kw. | 1,266 |
| 55 | (behavio* adj3 (reinforce* or re-inforce*)).ti,ab,kw. | 1,386 |
| 56 | (behavio* adj2 (contracting or modif*)).ti,ab,kw. | 4,325 |
| 57 | reinforc*.ti,kw. | 6,150 |
| 58 | ((positive adj1 reinforc*) or (reinforc* adj3 (environment* or experience*))).ti,ab,kw. | 1,478 |
| 59 | ((behavio* adj3 motivat*) or motivational interviewing).ti,ab,kw. | 8,734 |
| 60 | (activit* adj2 schedul*).ti,ab,kw. | 364 |
| 61 | ((pleas* or enjoyable or rewarding) adj3 (activit* or event?)).ti,ab,kw. | 904 |
| 62 | ((operant or instrumental) adj (conditioning or learning)).ti,ab,kw. | 672 |
| 63 | (positive interaction* or avoida* coping or environmental contingenc* or contingency management).ti,ab,kw. | 3,517 |
| 64 | functional analysis.ti,ab,kw. | 4,733 |
| 65 | behavio*.mp. and (self adj (evaluat* or monitor*)).ti,ab,kw. | 3,002 |
| 66 | (behavio* adj (counsel* or intervention* or train* or treatment* or therap* or psychotherap*)).ti,ab,kw. | 31,116 |
| 67 | (mood adj3 monitor*).ti,ab,kw. | 161 |
| 68 | or/51-67 | 70,858 |
| 69 | 50 and 68 | 2,103 |
| 70 | limit 69 to dc=20221025-20230330 | 63 |

**Table S3. Embase**

**Database:**
Embase Classic+Embase <1947 to 2023 March 28>

| **#** | **Query** | **Results from 30 Mar 2023** |
| --- | --- | --- |
| 1 | non communicable disease/ | 11,284 |
| 2 | ((non communicable or noncommunicable or non infectious or noninfectious) adj3 disease*).ti,ab,kf. | 24,051 |
| 3 | Chronic Disease/ | 224,144 |
| 4 | (chronic* adj3 (condition or conditions or disease* or ill*)).ti,ab,kf. | 631,351 |
| 5 | chronic obstructive lung disease/ | 171,931 |
| 6 | obstructive lung disease/ | 607 |
| 7 | exp Asthma/ | 318,267 |
| 8 | respiratory tract allergy/ | 12,772 |
| 9 | pulmonary hypertension/ | 105,337 |
| 10 | ((long term or longterm or chronic*) adj5 (bronchitis or respirat*)).ti,ab,kf. | 49,178 |
| 11 | emphysema*.ti,ab,kf. | 49,139 |
| 12 | asthma*.ti,ab,kf. | 280,745 |
| 13 | (obstruct* adj3 (pulmonary or lung* or airway* or airflow* or bronch* or respirat*)).ti,ab,kf. | 159,690 |
| 14 | ((hyper responsiveness or hyper-responsiveness or allergy or allergi* or hypersensitiv* or hyperreactiv* or insufficiency) adj5 (airway or respirat* or bronchial* or lung*)).ti,ab,kf. | 51,699 |
| 15 | ((longterm or long term or Chronic* or occupational or recur*) adj2 lung* adj5 (condition* or disease* or symptom* or problem* or failure* or ill*)).ti,ab,kf. | 28,344 |
| 16 | (respirat* adj2 (condition* or disease* or symptom* or problem* or failure or ill*)).ti,ab,kf. | 194,901 |
| 17 | pulmonary hypertension.ti,ab,kf. | 76,148 |
| 18 | (COPD or COAD or COBD or AECB or AECOPD).ti,ab,kf. | 111,967 |
| 19 | or/1-18 | 1,516,241 |
| 20 | exp Diabetes Mellitus/ | 1,250,946 |
| 21 | Glucose Tolerance Test/ | 34,041 |
| 22 | hemoglobin A1c/ | 145,914 |
| 23 | glucose blood level/ | 332,370 |
| 24 | diabet*.ti,ab,kf. | 1,215,681 |
| 25 | (noninsulin*-depend* or non-insulin*-depend* or noninsulin*depend* or non-insulin*depend*).ti,ab,kf. | 15,291 |
| 26 | (fasting glucose or plasma glucose or glucose tolerance test* or ((glycemic or glycaemic) adj2 control*)).ti,ab,kf. | 178,218 |
| 27 | (HbA1c or A1C or A1c or Hb1c or ((glycated or glycosylated) adj (hemoglobin or haemoglobin))).ti,ab,kf. | 135,027 |
| 28 | (NIDDM or T2D or T2DM or T1D or IDDM or MODY or T1DM).ti,ab,kf. | 128,866 |
| 29 | or/20-28 | 1,651,589 |
| 30 | exp cardiovascular disease/ | 5,321,005 |
| 31 | blood pressure/ | 323,825 |
| 32 | (arteriosclero* or atherosclero* or peripheral arter* disease* or blood pressure).ti,ab,kf. | 813,749 |
| 33 | (cardio* or cardia* or cvd or heart* or coronary or angina or ventric* or myocard* or pericard* or ischem* or ischaem* or emboli* or cerebrovasc* or cerebral vascular or arrhythmi* or thrombo* or atrial fibrillat* or angioplasty or infarct* or apoplexy or stroke or strokes or poststroke or hypertensi*).ti,ab,kf. | 5,202,221 |
| 34 | or/30-33 | 7,082,555 |
| 35 | exp neoplasm/ | 5,923,924 |
| 36 | (cancer* or neoplas* or tumo* or carcinoma* or hodgkin* or nonhodgkin* or non hodgkin* or adenocarcinoma* or leukemia* or leukaemia* or metasta* or malignan* or lymphoma* or sarcoma* or melanoma* or myeloma* or oncolog* or psychooncolog* or glioma*).ti,ab,kf. | 6,295,064 |
| 37 | 35 or 36 | 7,372,794 |
| 38 | 19 or 29 or 34 or 37 | 15,027,107 |
| 39 | depression/ | 475,964 |
| 40 | mood disorder/ | 52,494 |
| 41 | major depression/ | 80,890 |
| 42 | treatment resistant depression/ | 4,949 |
| 43 | dysthymia/ | 9,897 |
| 44 | cyclothymia/ | 1,179 |
| 45 | minor depression/ | 324 |
| 46 | post-stroke depression/ | 1,234 |
| 47 | subsyndromal depression/ | 495 |
| 48 | (depress* or dysthymi* or cyclothymi* or low mood or mood disorder* or affective disorder*).ti,ab,kf. | 824,821 |
| 49 | or/39-48 | 987,505 |
| 50 | 38 and 49 | 311,414 |
| 51 | Behavior Therapy/ | 47,122 |
| 52 | Motivational Interviewing/ | 6,743 |
| 53 | behavio* activat*.ti,ab,kf. | 3,221 |
| 54 | (behavio* activat* or BATD).ti,ab,kf. | 3,227 |
| 55 | (behavio* adj3 (reinforce* or re-inforce*)).ti,ab,kf. | 4,097 |
| 56 | (behavio* adj2 (contracting or modif*)).ti,ab,kf. | 15,085 |
| 57 | reinforc*.ti,kf. | 29,175 |
| 58 | ((positive adj1 reinforc*) or (reinforc* adj3 (environment* or experience*))).ti,ab,kf. | 4,689 |
| 59 | ((behavio* adj3 motivat*) or motivational interviewing).ti,ab,kf. | 18,126 |
| 60 | (activit* adj2 schedul*).ti,ab,kf. | 1,012 |
| 61 | ((pleas* or enjoyable or rewarding) adj3 (activit* or event?)).ti,ab,kf. | 2,097 |
| 62 | ((operant or instrumental) adj (conditioning or learning)).ti,ab,kf. | 5,097 |
| 63 | (positive interaction* or avoida* coping or environmental contingenc* or contingency management).ti,ab,kf. | 7,529 |
| 64 | functional analysis.ti,ab,kf. | 37,211 |
| 65 | behavio*.mp. and (self adj (evaluat* or monitor*)).ti,ab,kf. | 6,387 |
| 66 | (behavio* adj (counsel* or intervention* or train* or treatment* or therap* or psychotherap*)).ti,ab,kf. | 69,775 |
| 67 | (mood adj3 monitor*).ti,ab,kf. | 503 |
| 68 | or/51-67 | 220,107 |
| 69 | 50 and 68 | 6,672 |
| 70 | limit 69 to dc=20221025-20230330 | 285 |

**Table S4. APA PsycInfo**

**Database:**
APA PsycInfo <1806 to March Week 3 2023>

| **#** | **Query** | **Results from 30 Mar 2023** |
| --- | --- | --- |
| 1 | exp Chronic Illness/ | 33,612 |
| 2 | ((non communicable or noncommunicable or non infectious or noninfectious) adj3 disease*).ti,ab. | 1,422 |
| 3 | (chronic* adj3 (condition or conditions or disease* or ill*)).ti,ab. | 47,593 |
| 4 | exp Chronic Obstructive Pulmonary Disease/ | 1,735 |
| 5 | exp Asthma/ | 5,105 |
| 6 | Respiratory Distress/ | 688 |
| 7 | ((long term or longterm or chronic*) adj5 (bronchitis or respirat*)).ti,ab. | 784 |
| 8 | emphysema*.ti,ab. | 271 |
| 9 | asthma*.ti,ab. | 8,330 |
| 10 | (obstruct* adj3 (pulmonary or lung* or airway* or airflow* or bronch* or respirat*)).ti,ab. | 3,157 |
| 11 | ((hyper responsiveness or hyper-responsiveness or allergi* or hypersensitiv* or hyperreactiv* or insufficiency) adj5 (airway or respirat* or bronchial* or lung*)).ti,ab. | 396 |
| 12 | ((longterm or long term or Chronic* or occupational or recur*) adj2 lung* adj5 (condition* or disease* or symptom* or problem* or failure* or ill*)).ti,ab. | 445 |
| 13 | (respirat* adj2 (condition* or disease* or symptom* or problem* or failure or ill*)).ti,ab. | 3,535 |
| 14 | pulmonary hypertension.ti,ab. | 219 |
| 15 | (COPD or COAD or COBD or AECB or AECOPD).ti,ab. | 1,885 |
| 16 | 1 or 2 or 3 or 4 or 5 or 6 or 7 or 8 or 9 or 10 or 11 or 12 or 13 or 14 or 15 | 81,052 |
| 17 | exp Diabetes Mellitus/ or Diabetes/ or Type 2 Diabetes/ | 20,368 |
| 18 | Blood Sugar/ | 1,511 |
| 19 | diabet*.ti,ab. | 35,691 |
| 20 | (noninsulin*-depend* or non-insulin*-depend* or noninsulin*depend* or non-insulin*depend*).ti,ab. | 280 |
| 21 | (fasting glucose or plasma glucose or glucose tolerance test* or ((glycemic or glycaemic) adj2 control*)).ti,ab. | 4,439 |
| 22 | (HbA1c or A1C or A1c or Hb1c or ((glycated or glycosylated) adj (hemoglobin or haemoglobin))).ti,ab. | 3,675 |
| 23 | (NIDDM or T2D or T2DM or T1D or IDDM or MODY or T1DM).ti,ab. | 3,047 |
| 24 | 17 or 18 or 19 or 20 or 21 or 22 or 23 | 38,531 |
| 25 | exp Cardiovascular Disorders/ or Heart Disorders/ or Blood Pressure/ | 75,671 |
| 26 | (arteriosclero* or atherosclero* or peripheral arter* disease* or blood pressure).ti,ab. | 24,620 |
| 27 | (cardio* or cardia* or cvd or heart* or coronary or angina or ventric* or myocard* or pericard* or ischem* or ischaem* or emboli* or cerebrovasc* or cerebral vascular or arrhythmi* or thrombo* or atrial fibrillat* or infarct* or apoplexy or stroke or strokes or poststroke or hypertensi*).ti,ab. | 177,880 |
| 28 | 25 or 26 or 27 | 192,398 |
| 29 | exp Neoplasms/ | 60,105 |
| 30 | (cancer* or neoplas* or tumo* or carcinoma* or hodgkin* or nonhodgkin* or non hodgkin* or adenocarcinoma* or leukemia* or leukaemia* or metasta* or malignan* or lymphoma* or sarcoma* or melanoma* or myeloma* or oncolog* or psychooncolog* or glioma*).ti,ab. | 96,386 |
| 31 | 29 or 30 | 98,410 |
| 32 | 16 or 24 or 28 or 31 | 366,811 |
| 33 | "Depression (Emotion)"/ | 26,884 |
| 34 | Affective Disorders/ | 15,761 |
| 35 | Major Depression/ | 146,862 |
| 36 | Treatment Resistant Depression/ | 2,920 |
| 37 | Dysthymic Disorder/ | 1,523 |
| 38 | Cyclothymic Disorder/ | 228 |
| 39 | (depress* or dysthymi* or cyclothymi* or low mood or mood disorder* or affective disorder*).ti,ab. | 362,641 |
| 40 | 33 or 34 or 35 or 36 or 37 or 38 or 39 | 372,214 |
| 41 | 32 and 40 | 47,665 |
| 42 | Behavior Therapy/ or Behavioral Activation System/ | 16,461 |
| 43 | Motivational Interviewing/ | 3,014 |
| 44 | (behavio* activat* or BATD).ti,ab. | 2,570 |
| 45 | (behavio* adj3 (reinforce* or re-inforce*)).ti,ab. | 5,087 |
| 46 | (behavio* adj2 (contracting or modif*)).ti,ab. | 9,497 |
| 47 | Reinforcement/ | 14,659 |
| 48 | reinforc*.ti,ab. | 84,776 |
| 49 | (reinforce or reinforcer or reinforcement or reinforcements or re-inforcement or re-inforcements).ti,ab. | 59,256 |
| 50 | ((positive adj1 reinforc*) or (reinforc* adj3 (environment* or experience*))).ti,ab. | 4,875 |
| 51 | ((behavio* adj3 motivat*) or motivational interviewing).ti,ab. | 15,472 |
| 52 | (activit* adj2 schedul*).ti,ab. | 647 |
| 53 | ((pleas* or enjoyable or rewarding) adj3 (activit* or event or events)).ti,ab. | 1,936 |
| 54 | ((operant or instrumental) adj (conditioning or learning)).ti,ab. | 5,351 |
| 55 | (positive interaction* or avoida* coping or environmental contingenc* or contingency management).ti,ab. | 6,375 |
| 56 | Functional Analysis/ | 2,214 |
| 57 | functional analysis.ti,ab. | 3,230 |
| 58 | behavio*.mp. and (self adj (evaluat* or monitor*)).ti,ab. | 6,534 |
| 59 | (behavio* adj (counsel* or intervention* or train* or treatment* or therap* or psychotherap*)).ti,ab. | 58,187 |
| 60 | (mood adj3 monitor*).ti,ab. | 301 |
| 61 | 42 or 43 or 44 or 45 or 46 or 47 or 48 or 49 or 50 or 51 or 52 or 53 or 54 or 55 or 56 or 57 or 58 or 59 or 60 | 189,926 |
| 62 | 41 and 61 | 2,142 |
| 63 | limit 62 to up=20221025-20230330 | 45 |

**Table S5. Cochrane**

**Search Name: BAT and NCDs Final Cochrane search updated 30-3-20232**

**Date Run: 30/03/2023 05:53:34**

Comment:

ID Search Hits

#1 MeSH descriptor: [Noncommunicable Diseases] this term only 77

#2 ((("non communicable" or noncommunicable or "non infectious" or noninfectious) near/3 disease*)):ti,ab,kw 1051

#3 MeSH descriptor: [Chronic Disease] this term only 15169

#4 ((chronic* near/3 (condition or conditions or disease* or ill*))):ti,ab,kw 72079

#5 MeSH descriptor: [Pulmonary Disease, Chronic Obstructive] explode all trees 7184

#6 MeSH descriptor: [Lung Diseases, Obstructive] this term only 3298

#7 MeSH descriptor: [Asthma] explode all trees 13406

#8 MeSH descriptor: [Respiratory Hypersensitivity] this term only 253

#9 MeSH descriptor: [Hypertension, Pulmonary] this term only 1196

#10 ((("long term" or longterm or chronic*) near/5 (bronchitis or respirat*))):ti,ab,kw 5020

#11 (emphysema*):ti,ab,kw 1704

#12 (asthma*):ti,ab,kw 36887

#13 ((obstruct* near/3 (pulmonary or lung* or airway* or airflow* or bronch* or respirat*))):ti,ab,kw 22874

#14 ((("hyper responsiveness" or hyperresponsiveness or allergy or allergi* or hypersensitiv* or hyperreactiv* or insufficiency) near/5 (airway or respirat* or bronchial* or lung*))):ti,ab,kw 6314

#15 (((longterm or "long term" or Chronic* or occupational or recur*) near/2 lung* near/5 (condition* or disease* or symptom* or problem* or failure* or ill*))):ti,ab,kw 9281

#16 ((respirat* near/2 (condition* or disease* or symptom* or problem* or failure or ill*))):ti,ab,kw 17434

#17 ("pulmonary hypertension"):ti,ab,kw 3466

#18 ((COPD or COAD or COBD or AECB or AECOPD)):ti,ab,kw 18841

#19 {OR #1-#18} 131869

#20 MeSH descriptor: [Diabetes Mellitus] explode all trees 40123

#21 MeSH descriptor: [Diabetes Mellitus, Type 2] this term only 22820

#22 MeSH descriptor: [Diabetes Mellitus, Type 1] this term only 6706

#23 MeSH descriptor: [Glucose Tolerance Test] this term only 2341

#24 MeSH descriptor: [Glycated Hemoglobin] this term only 7181

#25 MeSH descriptor: [Blood Glucose] this term only 19202

#26 (diabet*):ti,ab,kw 111414

#27 ((noninsulin NEXT depend* or non NEXT insulin NEXT depend* or noninsulin NEXT depend* or non NEXT insulin depend*)):ti,ab,kw 20715

#28 (("fasting glucose" or "plasma glucose" or "glucose NEXT tolerance test*" or ((glycemic or glycaemic) near/2 control*))):ti,ab,kw 28038

#29 ((HbA1c or A1C or A1c or Hb1c or ((glycated or glycosylated) NEXT (hemoglobin or haemoglobin)))):ti,ab,kw 32274

#30 ((NIDDM or T2D or T2DM or T1D or IDDM or MODY or T1DM)):ti,ab,kw 16262

#31 {OR #20-#30} 126923

#32 MeSH descriptor: [Cardiovascular Diseases] explode all trees 135568

#33 MeSH descriptor: [Blood Pressure] this term only 29726

#34 ((arteriosclero* or atherosclero* or peripheral arter* NEXT disease* or "blood pressure")):ti,ab,kw 122684

#35 ((cardio* or cardia* or cvd or heart* or coronary or angina or ventric* or myocard* or pericard* or ischem* or ischaem* or emboli* or cerebrovasc* or "cerebral vascular" or arrhythmi* or thrombo* or atrial NEXT fibrillat* or infarct* or apoplexy or stroke or strokes or poststroke or hypertensi*)):ti,ab,kw 417998

#36 #32 or #33 or #34 or #35 458830

#37 MeSH descriptor: [Neoplasms] explode all trees 106545

#38 ((cancer* or neoplas* or tumo* or carcinoma* or hodgkin* or nonhodgkin* or "non hodgkin" or adenocarcinoma* or leukemia* or leukaemia* or metasta* or malignan* or lymphoma* or sarcoma* or melanoma* or myeloma* or oncolog* or psychooncolog* or glioma*)):ti,ab,kw 276370

#39 #37 OR #38 281147

#40 #19 OR #31 OR #36 OR #39 850093

#41 MeSH descriptor: [Depression] this term only 16000

#42 MeSH descriptor: [Mood Disorders] this term only 1009

#43 MeSH descriptor: [Depressive Disorder] this term only 9091

#44 MeSH descriptor: [Depressive Disorder, Major] this term only 6396

#45 MeSH descriptor: [Depressive Disorder, Treatment-Resistant] this term only 617

#46 MeSH descriptor: [Dysthymic Disorder] this term only 196

#47 MeSH descriptor: [Cyclothymic Disorder] this term only 18

#48 ((depress* or dysthymi* or cyclothymi* or "low mood" or "mood NEXT disorder*" or "affective NEXT disorder*")):ti,ab,kw 104920

#49 {OR #41-#48} 105404

#50 #40 AND #49 34512

#51 MeSH descriptor: [Behavior Therapy] this term only 5424

#52 MeSH descriptor: [Motivational Interviewing] this term only 1158

#53 (behavio* NEXT activat*):ti,ab,kw 1185

#54 ((behavio* NEXT activat* or BATD)):ti,ab,kw 1189

#55 ((behavio* near/3 (reinforce* or re NEXT inforce*))):ti,ab,kw 370

#56 ((behavio* near/2 (contracting or modif*))):ti,ab,kw 2329

#57 (reinforc*):ti,ab,kw 10215

#58 (((positive near/1 reinforc*) or (reinforc* near/3 (environment* or experience*)))):ti,ab,kw 430

#59 (((behavio* near/3 motivat*) or "motivational interviewing")):ti,ab,kw 5961

#60 ((activit* near/2 schedul*)):ti,ab,kw 323

#61 (((pleas* or enjoyable or rewarding) near/3 (activit* or event?))):ti,ab,kw 460

#62 (((operant or instrumental) near/1 (conditioning or learning))):ti,ab,kw 392

#63 (("positive NEXT interaction*" or avoida* coping or environmental NEXT contingenc* or "contingency management")):ti,ab,kw 1570

#64 ("functional analysis"):ti,ab,kw 265

#65 (behavio* (self near/1 (evaluat* or monitor*))):ti,ab,kw 2927

#66 ((behavio* near/1 (counsel* or intervention* or train* or treatment* or therap* or psychotherap*))):ti,ab,kw 37559

#67 ((mood near/3 monitor*)):ti,ab,kw 229

#68 {OR #51-#67} 56884

#69 #50 AND #68 3335

#70 #50 AND #68 with Cochrane Library publication date Between Oct 2022 and May 2023 115

**Table S6. CINAHL**

| S1 | MH ((MH “Noncommunicable Diseases” OR “Chronic Disease” OR “Pulmonary Disease, Chronic Obstructive+” OR “Lung Diseases, Obstructive” OR “Asthma+” OR “Respiratory Hypersensitivity” Or “Hypertension, Pulmonary”)) | 142,049 |
| --- | --- | --- |
| S2 | TI ( ((“non communicable” or noncommunicable or “non infectious” or noninfectious) N2 disease*) ) OR AB ( ((“non communicable” or noncommunicable or “non infectious” or noninfectious) N2 disease*) ) OR TI ( (chronic* N2 (condition or conditions or disease* or ill*)) ) OR AB ( (chronic* N2 (condition or conditions or disease* or ill*)) ) | **131157** |
| 3 | TI ( (((“long term” or longterm or chronic*) N4 (bronchitis or respirat*)) OR emphysema* OR asthma*) ) OR AB ( (((“long term” or longterm or chronic*) N4 (bronchitis or respirat*)) OR emphysema* OR asthma*) ) OR TI ( (obstruct* N2 (pulmonary or lung* or airway* or airflow* or bronch* or respirat*)) ) OR AB ( (obstruct* N2 (pulmonary or lung* or airway* or airflow* or bronch* or respirat*)) ) | 73332 |
| S4 | TI ( ((“hyper responsiveness” or hyperresponsiveness or allergi* or hypersensitiv* or hyperreactiv* or insufficiency) N4 (airway or respirat* or bronchial* or lung*)) ) OR AB ( ((“hyper responsiveness” or hyperresponsiveness or allergi* or hypersensitiv* or hyperreactiv* or insufficiency) N4 (airway or respirat* or bronchial* or lung*)) ) OR TI ( ((longterm or “long term” or Chronic* or occupational or recur*) N1 lung* N4 (condition* or disease* or symptom* or problem* or failure* or ill*)) ) OR AB ( ((longterm or “long term” or Chronic* or occupational or recur*) N1 lung* N4 (condition* or disease* or symptom* or problem* or failure* or ill*)) ) | 8,053 |
| S5 | TI ( (respirat* N1 (condition* or disease* or symptom* or problem* or failure or ill*)) ) OR AB ( (respirat* N1 (condition* or disease* or symptom* or problem* or failure or ill*)) ) OR TI ( (“pulmonary hypertension” OR COPD or COAD or COBD or AECB or AECOPD) ) OR AB ( (“pulmonary hypertension” OR COPD or COAD or COBD or AECB or AECOPD) ) | 62,083 |
| S6 | TI S1 OR S2 OR S3 OR S4 OR S5 | 275,992 |
| S7 | (MH (“Diabetes Mellitus+” OR “Diabetes Mellitus, Type 2” OR “Diabetes Mellitus, Type 1” OR “Glucose Tolerance Test” OR “Blood Glucose”)) | 211,280 |
| S8 | TI ( (diabet* OR (noninsulin*-depend* or non-insulin*-depend* or noninsulin*depend* or non-insulin*depend*) OR (“fasting glucose” or “plasma glucose” or “glucose tolerance test*” or ((glycemic or glycaemic) N1 control*)) ) OR AB ( (diabet* OR (noninsulin*-depend* or non-insulin*-depend* or noninsulin*depend* or non-insulin*depend*) OR (“fasting glucose” or “plasma glucose” or “glucose tolerance test*” or ((glycemic or glycaemic) N1 control*)) ) | 250,318 |
| S9 | TI ( ((HbA1c or A1C or A1c or Hb1c or ((glycated or glycosylated) W0 (hemoglobin or haemoglobin))) OR (NIDDM or T2D or T2DM or T1D or IDDM or MODY or T1DM)) ) OR AB ( ((HbA1c or A1C or A1c or Hb1c or ((glycated or glycosylated) W0 (hemoglobin or haemoglobin))) OR (NIDDM or T2D or T2DM or T1D or IDDM or MODY or T1DM)) ) | 83,345 |
| S10 | S7 OR S8 OR S9 | 303,401 |
| S11 | (MH (“Cardiovascular Diseases+” OR “Blood Pressure”) | 698,616 |
| S12 | TI ( (arteriosclero* or atherosclero* or “peripheral arter*” disease* or “blood pressure”) OR (cardio* or cardia* or cvd or heart* or coronary or angina or ventric* or myocard* or pericard* or ischem* or ischaem* or emboli* or cerebrovasc* or “cerebral vascular” or arrhythmi* or thrombo* or “atrial fibrillat*” or infarct* or apoplexy or stroke or strokes or poststroke or hypertensi*) ) OR AB ( (arteriosclero* or atherosclero* or “peripheral arter*” disease* or “blood pressure”) OR (cardio* or cardia* or cvd or heart* or coronary or angina or ventric* or myocard* or pericard* or ischem* or ischaem* or emboli* or cerebrovasc* or “cerebral vascular” or arrhythmi* or thrombo* or “atrial fibrillat*” or infarct* or apoplexy or stroke or strokes or poststroke or hypertensi*) ) | 875,970 |
| S13 | S12 OR S11 | 1,083,914 |
| S14 | MH (Neoplasms+) OR TI ( (cancer* or neoplas* or tumo* or carcinoma* or hodgkin* or nonhodgkin* or “non hodgkin*” or adenocarcinoma* or leukemia* or leukaemia* or metasta* or malignan* or lymphoma* or sarcoma* or melanoma* or myeloma* or oncolog* or psychooncolog* or glioma*) ) OR AB ( (cancer* or neoplas* or tumo* or carcinoma* or hodgkin* or nonhodgkin* or “non hodgkin*” or adenocarcinoma* or leukemia* or leukaemia* or metasta* or malignan* or lymphoma* or sarcoma* or melanoma* or myeloma* or oncolog* or psychooncolog* or glioma*) ) | 927,644 |
| S15 | ( (MH (Depression OR “Affective Disorders” OR “Dysthymic Disorder” OR “Cyclothymic Disorder”) ) OR TI ( (depress* or dysthymi* or cyclothymi* or “low mood” or “mood disorder*” or “affective disorder*”) ) OR AB ( (depress* or dysthymi* or cyclothymi* or “low mood” or “mood disorder*” or “affective disorder*”) ) | 222,612 |
| S16 | S6 OR S10 OR S13 OR S14 | 2,276,593 |
| S17 | S15 AND S16 | 54,418 |
| S18 | (MH (“Behavior Therapy” OR “Motivational Interviewing”)) | 17,466 |
| S19 | TI ( (("behavio* activat*") OR ("behavio* activat*" or BATD) OR (behavio* N2 (reinforce* or "re inforce*" OR reinforce)) OR (behavio* N1 (contracting or modif*)) OR reinforc*) ) OR AB ( (("behavio* activat*") OR ("behavio* activat*" or BATD) OR (behavio* N2 (reinforce* or "re inforce*" OR reinforce)) OR (behavio* N1 (contracting or modif*)) OR reinforc*) ) | 30,976 |
| S20 | TI ( (((positive N0 reinforc*) or (reinforc* N2 (environment* or experience*)) OR (behavio* N2 motivat*) or “motivational interviewing” OR (activit* N1 schedul*) OR ((pleas* or enjoyable or rewarding) N2 (activit* or event?))) ) OR AB ( (((positive N0 reinforc*) or (reinforc* N2 (environment* or experience*)) OR (behavio* N2 motivat*) or “motivational interviewing” OR (activit* N1 schedul*) OR ((pleas* or enjoyable or rewarding) N2 (activit* or event?))) ) | 8,796 |
| S21 | TI ( (((operant or instrumental) W0 (conditioning or learning)) OR (“positive interaction*” or avoida* “coping or environmental contingenc*” or “contingency management”)) ) OR AB ( (((operant or instrumental) W0 (conditioning or learning)) OR (“positive interaction*” or avoida* “coping or environmental contingenc*” or “contingency management”)) ) | 1,742 |
| S22 | TI ( ((“functional analysis” OR (behavio* W0 (counsel* or intervention* or train* or treatment* or therap* or psychotherap*)) OR (mood N2 monitor*)) ) OR AB ( ((“functional analysis” OR (behavio* W0 (counsel* or intervention* or train* or treatment* or therap* or psychotherap*)) OR (mood N2 monitor*)) ) | 26,334 |
| S23 | (TI(self W0 (evaluat* or monitor*)) OR AB(self W0 (evaluat* or monitor*))) AND behavio* | 2,208 |
| S24 | S18 OR S19 OR S20 OR S21 OR S22 OR S23 | 75,985 |
| S25 | S17 AND S24 | 1,624 |
| S26 | S17 AND S24 Limiters - Published Date: 20221001-20230431 | 38 |

**Search strategy for the updated search (30 March 2023 to 23 September 2024.**

**Table S1.** Ovid MEDLINE(R) ALL <1946 to September 23, 2024>

| **#** | **Query** | **Results from 25 Sep 2024** |
| --- | --- | --- |
| 1 | Noncommunicable Diseases/ | 3,705 |
| 2 | ((non communicable or noncommunicable or non infectious or noninfectious) adj3 disease*).ti,ab,kf. | 22,833 |
| 3 | Chronic Disease/ | 287,582 |
| 4 | (chronic* adj3 (condition or conditions or disease* or ill*)).ti,ab,kf. | 462,096 |
| 5 | exp Pulmonary Disease, Chronic Obstructive/ | 70,467 |
| 6 | Lung Diseases, Obstructive/ | 18,302 |
| 7 | exp Asthma/ | 146,697 |
| 8 | Respiratory Hypersensitivity/ | 10,031 |
| 9 | Hypertension, Pulmonary/ | 40,531 |
| 10 | ((long term or longterm or chronic*) adj5 (bronchitis or respirat*)).ti,ab,kf. | 33,222 |
| 11 | emphysema*.ti,ab,kf. | 32,273 |
| 12 | asthma*.ti,ab,kf. | 190,170 |
| 13 | (obstruct* adj3 (pulmonary or lung* or airway* or airflow* or bronch* or respirat*)).ti,ab,kf. | 112,056 |
| 14 | ((hyper responsiveness or hyper-responsiveness or allergi* or hypersensitiv* or hyperreactiv* or insufficiency) adj5 (airway or respirat* or bronchial* or lung*)).ti,ab,kf. | 32,118 |
| 15 | ((longterm or long term or Chronic* or occupational or recur*) adj2 lung* adj5 (condition* or disease* or symptom* or problem* or failure* or ill*)).ti,ab,kf. | 19,304 |
| 16 | (respirat* adj2 (condition* or disease* or symptom* or problem* or failure or ill*)).ti,ab,kf. | 136,938 |
| 17 | pulmonary hypertension.ti,ab,kf. | 46,923 |
| 18 | (COPD or COAD or COBD or AECB or AECOPD).ti,ab,kf. | 65,471 |
| 19 | 1 or 2 or 3 or 4 or 5 or 6 or 7 or 8 or 9 or 10 or 11 or 12 or 13 or 14 or 15 or 16 or 17 or 18 | 1,134,046 |
| 20 | exp Diabetes Mellitus/ | 534,415 |
| 21 | Diabetes Mellitus, Type 2/ or Diabetes Mellitus, Type 1/ | 255,462 |
| 22 | Glucose Tolerance Test/ | 37,267 |
| 23 | Glycated Hemoglobin A/ | 45,531 |
| 24 | Blood Glucose/ | 188,925 |
| 25 | diabet*.ti,ab,kf. | 838,830 |
| 26 | (noninsulin*-depend* or non-insulin*-depend* or noninsulin*depend* or non-insulin*depend*).ti,ab,kf. | 12,547 |
| 27 | (fasting glucose or plasma glucose or glucose tolerance test* or ((glycemic or glycaemic) adj2 control*)).ti,ab,kf. | 120,992 |
| 28 | (HbA1c or A1C or A1c or Hb1c or ((glycated or glycosylated) adj (hemoglobin or haemoglobin))).ti,ab,kf. | 78,504 |
| 29 | (NIDDM or T2D or T2DM or T1D or IDDM or MODY or T1DM).ti,ab,kf. | 87,238 |
| 30 | 20 or 21 or 22 or 23 or 24 or 25 or 26 or 27 or 28 or 29 | 1,016,053 |
| 31 | exp Cardiovascular Diseases/ | 2,818,230 |
| 32 | Blood Pressure/ | 296,661 |
| 33 | (arteriosclero* or atherosclero* or peripheral arter* disease* or blood pressure).ti,ab,kf. | 570,157 |
| 34 | (cardio* or cardia* or cvd or heart* or coronary or angina or ventric* or myocard* or pericard* or ischem* or ischaem* or emboli* or cerebrovasc* or cerebral vascular or arrhythmi* or thrombo* or atrial fibrillat* or infarct* or apoplexy or stroke or strokes or poststroke or hypertensi*).ti,ab,kf. | 3,740,724 |
| 35 | 31 or 32 or 33 or 34 | 4,737,940 |
| 36 | exp Neoplasms/ | 4,022,038 |
| 37 | (cancer* or neoplas* or tumo* or carcinoma* or hodgkin* or nonhodgkin* or non hodgkin* or adenocarcinoma* or leukemia* or leukaemia* or metasta* or malignan* or lymphoma* or sarcoma* or melanoma* or myeloma* or oncolog* or psychooncolog* or glioma*).ti,ab,kf. | 4,783,419 |
| 38 | 36 or 37 | 5,529,768 |
| 39 | 19 or 30 or 35 or 38 | 11,031,970 |
| 40 | Depression/ | 161,877 |
| 41 | Mood Disorders/ | 16,379 |
| 42 | Depressive Disorder/ | 75,569 |
| 43 | Depressive Disorder, Major/ | 41,194 |
| 44 | Depressive Disorder, Treatment-Resistant/ | 2,428 |
| 45 | Dysthymic Disorder/ | 1,186 |
| 46 | Cyclothymic Disorder/ | 801 |
| 47 | (depress* or dysthymi* or cyclothymi* or low mood or mood disorder* or affective disorder*).ti,ab,kf. | 629,758 |
| 48 | 40 or 41 or 42 or 43 or 44 or 45 or 46 or 47 | 675,161 |
| 49 | 39 and 48 | 172,803 |
| 50 | Behavior Therapy/ | 31,086 |
| 51 | Motivational Interviewing/ | 2,758 |
| 52 | behavio* activat*.ti,ab,kf. | 2,909 |
| 53 | (behavio* activat* or BATD).ti,ab,kf. | 2,916 |
| 54 | (behavio* adj3 (reinforce* or re-inforce*)).ti,ab,kf. | 3,779 |
| 55 | (behavio* adj2 (contracting or modif*)).ti,ab,kf. | 12,178 |
| 56 | reinforc*.ti,kf. | 33,062 |
| 57 | ((positive adj1 reinforc*) or (reinforc* adj3 (environment* or experience*))).ti,ab,kf. | 3,894 |
| 58 | ((behavio* adj3 motivat*) or motivational interviewing).ti,ab,kf. | 15,500 |
| 59 | (activit* adj2 schedul*).ti,ab,kf. | 752 |
| 60 | ((pleas* or enjoyable or rewarding) adj3 (activit* or event?)).ti,ab,kf. | 1,636 |
| 61 | ((operant or instrumental) adj (conditioning or learning)).ti,ab,kf. | 3,942 |
| 62 | (positive interaction* or avoida* coping or environmental contingenc* or contingency management).ti,ab,kf. | 6,781 |
| 63 | functional analysis.ti,ab,kf. | 32,972 |
| 64 | behavio*.mp. and (self adj (evaluat* or monitor*)).ti,ab,kf. | 5,290 |
| 65 | (behavio* adj (counsel* or intervention* or train* or treatment* or therap* or psychotherap*)).ti,ab,kf. | 54,982 |
| 66 | (mood adj3 monitor*).ti,ab,kf. | 374 |
| 67 | 50 or 51 or 52 or 53 or 54 or 55 or 56 or 57 or 58 or 59 or 60 or 61 or 62 or 63 or 64 or 65 or 66 | 187,995 |
| 68 | 49 and 67 | 3,461 |
| 69 | limit 68 to dt=20230330-20240925 | 361 |

**Table S2. Ovid Emcare <1995 to 2024 Week 38>**

| **#** | **Query** | **Results from 25 Sep 2024** |
| --- | --- | --- |
| 1 | non communicable disease/ | 8,162 |
| 2 | ((non communicable or noncommunicable or non infectious or noninfectious) adj3 disease*).ti,ab,kw. | 11,889 |
| 3 | Chronic Disease/ | 72,274 |
| 4 | (chronic* adj3 (condition or conditions or disease* or ill*)).ti,ab,kw. | 163,907 |
| 5 | chronic obstructive lung disease/ | 49,914 |
| 6 | obstructive lung disease/ | 359 |
| 7 | exp Asthma/ | 68,122 |
| 8 | respiratory tract allergy/ | 1,737 |
| 9 | pulmonary hypertension/ | 22,894 |
| 10 | ((long term or longterm or chronic*) adj5 (bronchitis or respirat*)).ti,ab,kw. | 8,358 |
| 11 | emphysema*.ti,ab,kw. | 7,595 |
| 12 | asthma*.ti,ab,kw. | 51,600 |
| 13 | (obstruct* adj3 (pulmonary or lung* or airway* or airflow* or bronch* or respirat*)).ti,ab,kw. | 35,288 |
| 14 | ((hyper responsiveness or hyper-responsiveness or allergi* or hypersensitiv* or hyperreactiv* or insufficiency) adj5 (airway or respirat* or bronchial* or lung*)).ti,ab,kw. | 5,516 |
| 15 | ((longterm or long term or Chronic* or occupational or recur*) adj2 lung* adj5 (condition* or disease* or symptom* or problem* or failure* or ill*)).ti,ab,kw. | 5,571 |
| 16 | (respirat* adj2 (condition* or disease* or symptom* or problem* or failure or ill*)).ti,ab,kw. | 41,910 |
| 17 | pulmonary hypertension.ti,ab,kw. | 12,810 |
| 18 | (COPD or COAD or COBD or AECB or AECOPD).ti,ab,kw. | 22,179 |
| 19 | or/1-18 | 354,949 |
| 20 | exp Diabetes Mellitus/ | 330,387 |
| 21 | Glucose Tolerance Test/ | 4,824 |
| 22 | hemoglobin A1c/ | 49,351 |
| 23 | glucose blood level/ | 88,233 |
| 24 | diabet*.ti,ab,kw. | 284,114 |
| 25 | (noninsulin*-depend* or non-insulin*-depend* or noninsulin*depend* or non-insulin*depend*).ti,ab,kw. | 2,361 |
| 26 | (fasting glucose or plasma glucose or glucose tolerance test* or ((glycemic or glycaemic) adj2 control*)).ti,ab,kw. | 44,514 |
| 27 | (HbA1c or A1C or A1c or Hb1c or ((glycated or glycosylated) adj (hemoglobin or haemoglobin))).ti,ab,kw. | 35,394 |
| 28 | (NIDDM or T2D or T2DM or T1D or IDDM or MODY or T1DM).ti,ab,kw. | 31,715 |
| 29 | or/20-28 | 417,228 |
| 30 | exp cardiovascular disease/ | 1,236,346 |
| 31 | blood pressure/ | 65,700 |
| 32 | (arteriosclero* or atherosclero* or peripheral arter* disease* or blood pressure).ti,ab,kw. | 158,254 |
| 33 | (cardio* or cardia* or cvd or heart* or coronary or angina or ventric* or myocard* or pericard* or ischem* or ischaem* or emboli* or cerebrovasc* or cerebral vascular or arrhythmi* or thrombo* or atrial fibrillat* or infarct* or apoplexy or stroke or strokes or poststroke or hypertensi*).ti,ab,kw. | 1,078,629 |
| 34 | or/30-33 | 1,560,445 |
| 35 | exp neoplasm/ | 986,258 |
| 36 | (cancer* or neoplas* or tumo* or carcinoma* or hodgkin* or nonhodgkin* or non hodgkin* or adenocarcinoma* or leukemia* or leukaemia* or metasta* or malignan* or lymphoma* or sarcoma* or melanoma* or myeloma* or oncolog* or psychooncolog* or glioma*).ti,ab,kw. | 1,006,404 |
| 37 | 35 or 36 | 1,211,218 |
| 38 | 19 or 29 or 34 or 37 | 2,934,345 |
| 39 | depression/ | 172,826 |
| 40 | mood disorder/ | 20,082 |
| 41 | major depression/ | 31,160 |
| 42 | treatment resistant depression/ | 1,425 |
| 43 | dysthymia/ | 3,700 |
| 44 | cyclothymia/ | 439 |
| 45 | minor depression/ | 172 |
| 46 | post-stroke depression/ | 558 |
| 47 | subsyndromal depression/ | 287 |
| 48 | (depress* or dysthymi* or cyclothymi* or low mood or mood disorder* or affective disorder*).ti,ab,kw. | 255,900 |
| 49 | or/39-48 | 315,709 |
| 50 | 38 and 49 | 91,179 |
| 51 | Behavior Therapy/ | 17,591 |
| 52 | Motivational Interviewing/ | 4,473 |
| 53 | behavio* activat*.ti,ab,kw. | 1,467 |
| 54 | (behavio* activat* or BATD).ti,ab,kw. | 1,470 |
| 55 | (behavio* adj3 (reinforce* or re-inforce*)).ti,ab,kw. | 1,492 |
| 56 | (behavio* adj2 (contracting or modif*)).ti,ab,kw. | 4,783 |
| 57 | reinforc*.ti,kw. | 7,038 |
| 58 | ((positive adj1 reinforc*) or (reinforc* adj3 (environment* or experience*))).ti,ab,kw. | 1,652 |
| 59 | ((behavio* adj3 motivat*) or motivational interviewing).ti,ab,kw. | 9,982 |
| 60 | (activit* adj2 schedul*).ti,ab,kw. | 402 |
| 61 | ((pleas* or enjoyable or rewarding) adj3 (activit* or event?)).ti,ab,kw. | 1,018 |
| 62 | ((operant or instrumental) adj (conditioning or learning)).ti,ab,kw. | 718 |
| 63 | (positive interaction* or avoida* coping or environmental contingenc* or contingency management).ti,ab,kw. | 3,949 |
| 64 | functional analysis.ti,ab,kw. | 5,339 |
| 65 | behavio*.mp. and (self adj (evaluat* or monitor*)).ti,ab,kw. | 3,422 |
| 66 | (behavio* adj (counsel* or intervention* or train* or treatment* or therap* or psychotherap*)).ti,ab,kw. | 34,734 |
| 67 | (mood adj3 monitor*).ti,ab,kw. | 175 |
| 68 | or/51-67 | 81,918 |
| 69 | 50 and 68 | 2,772 |
| 70 | limit 69 to dc=20230330-20240925 | 309 |

**Table S3. Embase <1974 to 2024 September 23>**

| **#** | **Query** | **Results from 25 Sep 2024** |
| --- | --- | --- |
| 1 | non communicable disease/ | 13,475 |
| 2 | ((non communicable or noncommunicable or non infectious or noninfectious) adj3 disease*).ti,ab,kf. | 28,174 |
| 3 | Chronic Disease/ | 212,683 |
| 4 | (chronic* adj3 (condition or conditions or disease* or ill*)).ti,ab,kf. | 682,826 |
| 5 | chronic obstructive lung disease/ | 190,481 |
| 6 | obstructive lung disease/ | 1,369 |
| 7 | exp Asthma/ | 317,619 |
| 8 | respiratory tract allergy/ | 12,112 |
| 9 | pulmonary hypertension/ | 109,125 |
| 10 | ((long term or longterm or chronic*) adj5 (bronchitis or respirat*)).ti,ab,kf. | 46,065 |
| 11 | emphysema*.ti,ab,kf. | 42,555 |
| 12 | asthma*.ti,ab,kf. | 279,921 |
| 13 | (obstruct* adj3 (pulmonary or lung* or airway* or airflow* or bronch* or respirat*)).ti,ab,kf. | 165,765 |
| 14 | ((hyper responsiveness or hyper-responsiveness or allergy or allergi* or hypersensitiv* or hyperreactiv* or insufficiency) adj5 (airway or respirat* or bronchial* or lung*)).ti,ab,kf. | 48,797 |
| 15 | ((longterm or long term or Chronic* or occupational or recur*) adj2 lung* adj5 (condition* or disease* or symptom* or problem* or failure* or ill*)).ti,ab,kf. | 29,305 |
| 16 | (respirat* adj2 (condition* or disease* or symptom* or problem* or failure or ill*)).ti,ab,kf. | 205,977 |
| 17 | pulmonary hypertension.ti,ab,kf. | 78,441 |
| 18 | (COPD or COAD or COBD or AECB or AECOPD).ti,ab,kf. | 123,139 |
| 19 | or/1-18 | 1,565,034 |
| 20 | exp Diabetes Mellitus/ | 1,299,997 |
| 21 | Glucose Tolerance Test/ | 28,276 |
| 22 | hemoglobin A1c/ | 168,246 |
| 23 | glucose blood level/ | 339,053 |
| 24 | diabet*.ti,ab,kf. | 1,274,663 |
| 25 | (noninsulin*-depend* or non-insulin*-depend* or noninsulin*depend* or non-insulin*depend*).ti,ab,kf. | 15,436 |
| 26 | (fasting glucose or plasma glucose or glucose tolerance test* or ((glycemic or glycaemic) adj2 control*)).ti,ab,kf. | 187,164 |
| 27 | (HbA1c or A1C or A1c or Hb1c or ((glycated or glycosylated) adj (hemoglobin or haemoglobin))).ti,ab,kf. | 149,954 |
| 28 | (NIDDM or T2D or T2DM or T1D or IDDM or MODY or T1DM).ti,ab,kf. | 146,135 |
| 29 | or/20-28 | 1,733,478 |
| 30 | exp cardiovascular disease/ | 5,317,400 |
| 31 | blood pressure/ | 297,923 |
| 32 | (arteriosclero* or atherosclero* or peripheral arter* disease* or blood pressure).ti,ab,kf. | 810,477 |
| 33 | (cardio* or cardia* or cvd or heart* or coronary or angina or ventric* or myocard* or pericard* or ischem* or ischaem* or emboli* or cerebrovasc* or cerebral vascular or arrhythmi* or thrombo* or atrial fibrillat* or angioplasty or infarct* or apoplexy or stroke or strokes or poststroke or hypertensi*).ti,ab,kf. | 5,195,121 |
| 34 | or/30-33 | 7,062,638 |
| 35 | exp neoplasm/ | 5,862,771 |
| 36 | (cancer* or neoplas* or tumo* or carcinoma* or hodgkin* or nonhodgkin* or non hodgkin* or adenocarcinoma* or leukemia* or leukaemia* or metasta* or malignan* or lymphoma* or sarcoma* or melanoma* or myeloma* or oncolog* or psychooncolog* or glioma*).ti,ab,kf. | 6,374,251 |
| 37 | 35 or 36 | 7,353,060 |
| 38 | 19 or 29 or 34 or 37 | 14,950,903 |
| 39 | depression/ | 528,972 |
| 40 | mood disorder/ | 56,689 |
| 41 | major depression/ | 88,769 |
| 42 | treatment resistant depression/ | 6,026 |
| 43 | dysthymia/ | 9,555 |
| 44 | cyclothymia/ | 1,132 |
| 45 | minor depression/ | 357 |
| 46 | post-stroke depression/ | 1,514 |
| 47 | subsyndromal depression/ | 593 |
| 48 | (depress* or dysthymi* or cyclothymi* or low mood or mood disorder* or affective disorder*).ti,ab,kf. | 847,782 |
| 49 | or/39-48 | 1,025,771 |
| 50 | 38 and 49 | 326,437 |
| 51 | Behavior Therapy/ | 47,443 |
| 52 | Motivational Interviewing/ | 7,630 |
| 53 | behavio* activat*.ti,ab,kf. | 3,538 |
| 54 | (behavio* activat* or BATD).ti,ab,kf. | 3,546 |
| 55 | (behavio* adj3 (reinforce* or re-inforce*)).ti,ab,kf. | 4,128 |
| 56 | (behavio* adj2 (contracting or modif*)).ti,ab,kf. | 15,652 |
| 57 | reinforc*.ti,kf. | 30,383 |
| 58 | ((positive adj1 reinforc*) or (reinforc* adj3 (environment* or experience*))).ti,ab,kf. | 4,889 |
| 59 | ((behavio* adj3 motivat*) or motivational interviewing).ti,ab,kf. | 19,862 |
| 60 | (activit* adj2 schedul*).ti,ab,kf. | 1,073 |
| 61 | ((pleas* or enjoyable or rewarding) adj3 (activit* or event?)).ti,ab,kf. | 2,244 |
| 62 | ((operant or instrumental) adj (conditioning or learning)).ti,ab,kf. | 4,747 |
| 63 | (positive interaction* or avoida* coping or environmental contingenc* or contingency management).ti,ab,kf. | 8,212 |
| 64 | functional analysis.ti,ab,kf. | 39,997 |
| 65 | behavio*.mp. and (self adj (evaluat* or monitor*)).ti,ab,kf. | 6,880 |
| 66 | (behavio* adj (counsel* or intervention* or train* or treatment* or therap* or psychotherap*)).ti,ab,kf. | 75,498 |
| 67 | (mood adj3 monitor*).ti,ab,kf. | 536 |
| 68 | or/51-67 | 233,362 |
| 69 | 50 and 68 | 7,354 |
| 70 | limit 69 to dc=20230330-20240925 | 790 |

**Table S4. APA PsycInfo <1806 to September 2024 Week 3>**

| **#** | **Query** | **Results from 25 Sep 2024** |
| --- | --- | --- |
| 1 | exp Chronic Illness/ | 36,877 |
| 2 | ((non communicable or noncommunicable or non infectious or noninfectious) adj3 disease*).ti,ab. | 1,675 |
| 3 | (chronic* adj3 (condition or conditions or disease* or ill*)).ti,ab. | 51,561 |
| 4 | exp Chronic Obstructive Pulmonary Disease/ | 1,904 |
| 5 | exp Asthma/ | 5,474 |
| 6 | Respiratory Distress/ | 732 |
| 7 | ((long term or longterm or chronic*) adj5 (bronchitis or respirat*)).ti,ab. | 856 |
| 8 | emphysema*.ti,ab. | 277 |
| 9 | asthma*.ti,ab. | 8,827 |
| 10 | (obstruct* adj3 (pulmonary or lung* or airway* or airflow* or bronch* or respirat*)).ti,ab. | 3,397 |
| 11 | ((hyper responsiveness or hyper-responsiveness or allergi* or hypersensitiv* or hyperreactiv* or insufficiency) adj5 (airway or respirat* or bronchial* or lung*)).ti,ab. | 407 |
| 12 | ((longterm or long term or Chronic* or occupational or recur*) adj2 lung* adj5 (condition* or disease* or symptom* or problem* or failure* or ill*)).ti,ab. | 468 |
| 13 | (respirat* adj2 (condition* or disease* or symptom* or problem* or failure or ill*)).ti,ab. | 3,849 |
| 14 | pulmonary hypertension.ti,ab. | 231 |
| 15 | (COPD or COAD or COBD or AECB or AECOPD).ti,ab. | 2,055 |
| 16 | 1 or 2 or 3 or 4 or 5 or 6 or 7 or 8 or 9 or 10 or 11 or 12 or 13 or 14 or 15 | 87,934 |
| 17 | exp Diabetes Mellitus/ or Diabetes/ or Type 2 Diabetes/ | 21,312 |
| 18 | Blood Sugar/ | 1,678 |
| 19 | diabet*.ti,ab. | 38,355 |
| 20 | (noninsulin*-depend* or non-insulin*-depend* or noninsulin*depend* or non-insulin*depend*).ti,ab. | 282 |
| 21 | (fasting glucose or plasma glucose or glucose tolerance test* or ((glycemic or glycaemic) adj2 control*)).ti,ab. | 4,719 |
| 22 | (HbA1c or A1C or A1c or Hb1c or ((glycated or glycosylated) adj (hemoglobin or haemoglobin))).ti,ab. | 4,021 |
| 23 | (NIDDM or T2D or T2DM or T1D or IDDM or MODY or T1DM).ti,ab. | 3,515 |
| 24 | 17 or 18 or 19 or 20 or 21 or 22 or 23 | 41,416 |
| 25 | exp Cardiovascular Disorders/ or Heart Disorders/ or Blood Pressure/ | 80,648 |
| 26 | (arteriosclero* or atherosclero* or peripheral arter* disease* or blood pressure).ti,ab. | 25,898 |
| 27 | (cardio* or cardia* or cvd or heart* or coronary or angina or ventric* or myocard* or pericard* or ischem* or ischaem* or emboli* or cerebrovasc* or cerebral vascular or arrhythmi* or thrombo* or atrial fibrillat* or infarct* or apoplexy or stroke or strokes or poststroke or hypertensi*).ti,ab. | 188,670 |
| 28 | 25 or 26 or 27 | 203,909 |
| 29 | exp Neoplasms/ | 65,358 |
| 30 | (cancer* or neoplas* or tumo* or carcinoma* or hodgkin* or nonhodgkin* or non hodgkin* or adenocarcinoma* or leukemia* or leukaemia* or metasta* or malignan* or lymphoma* or sarcoma* or melanoma* or myeloma* or oncolog* or psychooncolog* or glioma*).ti,ab. | 103,383 |
| 31 | 29 or 30 | 105,489 |
| 32 | 16 or 24 or 28 or 31 | 391,673 |
| 33 | "Depression (Emotion)"/ | 27,302 |
| 34 | Affective Disorders/ | 16,661 |
| 35 | Major Depression/ | 163,153 |
| 36 | Treatment Resistant Depression/ | 3,374 |
| 37 | Dysthymic Disorder/ | 1,544 |
| 38 | Cyclothymic Disorder/ | 232 |
| 39 | (depress* or dysthymi* or cyclothymi* or low mood or mood disorder* or affective disorder*).ti,ab. | 391,040 |
| 40 | 33 or 34 or 35 or 36 or 37 or 38 or 39 | 401,023 |
| 41 | 32 and 40 | 51,712 |
| 42 | Behavior Therapy/ or Behavioral Activation System/ | 17,337 |
| 43 | Motivational Interviewing/ | 3,255 |
| 44 | (behavio* activat* or BATD).ti,ab. | 2,830 |
| 45 | (behavio* adj3 (reinforce* or re-inforce*)).ti,ab. | 5,307 |
| 46 | (behavio* adj2 (contracting or modif*)).ti,ab. | 9,809 |
| 47 | Reinforcement/ | 15,303 |
| 48 | reinforc*.ti,ab. | 89,361 |
| 49 | (reinforce or reinforcer or reinforcement or reinforcements or re-inforcement or re-inforcements).ti,ab. | 62,068 |
| 50 | ((positive adj1 reinforc*) or (reinforc* adj3 (environment* or experience*))).ti,ab. | 5,101 |
| 51 | ((behavio* adj3 motivat*) or motivational interviewing).ti,ab. | 16,745 |
| 52 | (activit* adj2 schedul*).ti,ab. | 684 |
| 53 | ((pleas* or enjoyable or rewarding) adj3 (activit* or event or events)).ti,ab. | 2,090 |
| 54 | ((operant or instrumental) adj (conditioning or learning)).ti,ab. | 5,489 |
| 55 | (positive interaction* or avoida* coping or environmental contingenc* or contingency management).ti,ab. | 6,863 |
| 56 | Functional Analysis/ | 2,323 |
| 57 | functional analysis.ti,ab. | 3,372 |
| 58 | behavio*.mp. and (self adj (evaluat* or monitor*)).ti,ab. | 6,902 |
| 59 | (behavio* adj (counsel* or intervention* or train* or treatment* or therap* or psychotherap*)).ti,ab. | 62,402 |
| 60 | (mood adj3 monitor*).ti,ab. | 324 |
| 61 | 42 or 43 or 44 or 45 or 46 or 47 or 48 or 49 or 50 or 51 or 52 or 53 or 54 or 55 or 56 or 57 or 58 or 59 or 60 | 201,619 |
| 62 | 41 and 61 | 2,364 |
| 63 | limit 62 to up=20230330-20240925 | 213 |

**Table S5. Cochrane search**

Search Name: **Cochrane search NCD 25-9-2024**

Date Run: 25/09/2024 02:46:37

Comment:

ID Search Hits

#1 MeSH descriptor: [Noncommunicable Diseases] explode all trees 115

#2 (((("non communicable" or noncommunicable or "non infectious" or noninfectious) near/3 disease*))):ti,ab,kw 1328

#3 MeSH descriptor: [Chronic Disease] this term only 16792

#4 (((chronic* near/3 (condition or conditions or disease* or ill*)))):ti,ab,kw 82235

#5 MeSH descriptor: [Pulmonary Disease, Chronic Obstructive] explode all trees 8331

#6 MeSH descriptor: [Lung Diseases, Obstructive] this term only 3483

#7 MeSH descriptor: [Asthma] explode all trees 15089

#8 MeSH descriptor: [Respiratory Hypersensitivity] this term only 285

#9 MeSH descriptor: [Hypertension, Pulmonary] this term only 1392

#10 (((("long term" or longterm or chronic*) near/5 (bronchitis or respirat*)))):ti,ab,kw 5467

#11 ((emphysema*)):ti,ab,kw 1869

#12 ((asthma*)):ti,ab,kw 38837

#13 (((obstruct* near/3 (pulmonary or lung* or airway* or airflow* or bronch* or respirat*)))):ti,ab,kw 25029

#14 (((("hyper responsiveness" or hyperresponsiveness or allergy or allergi* or hypersensitiv* or hyperreactiv* or insufficiency) near/5 (airway or respirat* or bronchial* or lung*)))):ti,ab,kw 6980

#15 ((((longterm or "long term" or Chronic* or occupational or recur*) near/2 lung* near/5 (condition* or disease* or symptom* or problem* or failure* or ill*)))):ti,ab,kw 10561

#16 (((respirat* near/2 (condition* or disease* or symptom* or problem* or failure or ill*)))):ti,ab,kw 19664

#17 (("pulmonary hypertension")):ti,ab,kw 3822

#18 (((COPD or COAD or COBD or AECB or AECOPD))):ti,ab,kw 20450

#19 {OR #1-#18} 146052

#20 MeSH descriptor: [Diabetes Mellitus] explode all trees 46530

#21 MeSH descriptor: [Diabetes Mellitus, Type 2] this term only 26590

#22 MeSH descriptor: [Diabetes Mellitus, Type 1] this term only 7653

#23 MeSH descriptor: [Glucose Tolerance Test] this term only 2649

#24 MeSH descriptor: [Glycated Hemoglobin] this term only 8532

#25 MeSH descriptor: [Blood Glucose] this term only 22537

#26 ((diabet*)):ti,ab,kw 124968

#27 (((noninsulin NEXT depend* or non NEXT insulin NEXT depend* or noninsulin NEXT depend* or non NEXT insulin depend*))):ti,ab,kw 24014

#28 ((("fasting glucose" or "plasma glucose" or "glucose NEXT tolerance test*" or ((glycemic or glycaemic) near/2 control*)))):ti,ab,kw 31337

#29 (((HbA1c or A1C or A1c or Hb1c or ((glycated or glycosylated) NEXT (hemoglobin or haemoglobin))))):ti,ab,kw 37346

#30 (((NIDDM or T2D or T2DM or T1D or IDDM or MODY or T1DM))):ti,ab,kw 19340

#31 {OR #20-#30} 142711

#32 MeSH descriptor: [Cardiovascular Diseases] explode all trees 157319

#33 MeSH descriptor: [Blood Pressure] this term only 33628

#34 (((arteriosclero* or atherosclero* or peripheral arter* NEXT disease* or "blood pressure"))):ti,ab,kw 137314

#35 (((cardio* or cardia* or cvd or heart* or coronary or angina or ventric* or myocard* or pericard* or ischem* or ischaem* or emboli* or cerebrovasc* or "cerebral vascular" or arrhythmi* or thrombo* or atrial NEXT fibrillat* or infarct* or apoplexy or stroke or strokes or poststroke or hypertensi*))):ti,ab,kw 462044

#36 #32 or #33 or #34 or #35 508029

#37 MeSH descriptor: [Neoplasms] explode all trees 126379

#38 (((cancer* or neoplas* or tumo* or carcinoma* or hodgkin* or nonhodgkin* or "non hodgkin" or adenocarcinoma* or leukemia* or leukaemia* or metasta* or malignan* or lymphoma* or sarcoma* or melanoma* or myeloma* or oncolog* or psychooncolog* or glioma*))):ti,ab,kw 305465

#39 #37 OR #38 310991

#40 #19 OR #31 OR #36 OR #39 940431

#41 MeSH descriptor: [Depression] this term only 18552

#42 MeSH descriptor: [Mood Disorders] this term only 1152

#43 MeSH descriptor: [Depressive Disorder] this term only 9858

#44 MeSH descriptor: [Depressive Disorder, Major] this term only 7261

#45 MeSH descriptor: [Depressive Disorder, Treatment-Resistant] this term only 772

#46 MeSH descriptor: [Dysthymic Disorder] this term only 201

#47 MeSH descriptor: [Cyclothymic Disorder] this term only 25

#48 (((depress* or dysthymi* or cyclothymi* or "low mood" or "mood NEXT disorder*" or "affective NEXT disorder*"))):ti,ab,kw 120581

#49 {OR #41-#48} 121124

#50 #40 AND #49 39941

#51 MeSH descriptor: [Behavior Therapy] this term only 6030

#52 MeSH descriptor: [Motivational Interviewing] this term only 1373

#53 ((behavio* NEXT activat*)):ti,ab,kw 1480

#54 (((behavio* NEXT activat* or BATD))):ti,ab,kw 1484

#55 (((behavio* near/3 (reinforce* or re NEXT inforce*)))):ti,ab,kw 418

#56 (((behavio* near/2 (contracting or modif*)))):ti,ab,kw 2586

#57 ((reinforc*)):ti,ab,kw 11475

#58 ((((positive near/1 reinforc*) or (reinforc* near/3 (environment* or experience*))))):ti,ab,kw 502

#59 ((((behavio* near/3 motivat*) or "motivational interviewing"))):ti,ab,kw 6837

#60 (((activit* near/2 schedul*))):ti,ab,kw 381

#61 ((((pleas* or enjoyable or rewarding) near/3 (activit* or event?)))):ti,ab,kw 555

#62 ((((operant or instrumental) near/1 (conditioning or learning)))):ti,ab,kw 434

#63 ((("positive NEXT interaction*" or avoida* coping or environmental NEXT contingenc* or "contingency management"))):ti,ab,kw 1763

#64 (("functional analysis")):ti,ab,kw 311

#65 ((behavio* (self near/1 (evaluat* or monitor*)))):ti,ab,kw 3358

#66 (((behavio* near/1 (counsel* or intervention* or train* or treatment* or therap* or psychotherap*)))):ti,ab,kw 42646

#67 (((mood near/3 monitor*))):ti,ab,kw 273

#68 {OR #51-#67} 64655

#69 #50 AND #68 3863

#70 #50 AND #68 with Cochrane Library publication date Between January 2023 and September 2024 2

**Table S6. CINAHL 25-9-2024**

| [**Search ID#**](javascript:__doPostBack('ctl00$ctl00$MainContentArea$MainContentArea$historyControl$ReorderHistoryLink','')) | | **Search Terms** | **Results** |
| --- | --- | --- | --- |
|  | S26 | S17 AND S24 Limiters - Published Date: 20230431-20240925 | 33 |
|  | S25 | S17 AND S24 | 1702 |
|  | S24 | S18 OR S19 OR S20 OR S21 OR S22 OR S23 | 79511 |
|  | S23 | (TI(self W0 (evaluat* or monitor*)) OR AB(self W0 (evaluat* or monitor*))) AND behavio* | 2326 |
|  | S22 | TI ( ((“functional analysis” OR (behavio* W0 (counsel* or intervention* or train* or treatment* or therap* or psychotherap*)) OR (mood N2 monitor*)) ) OR AB ( ((“functional analysis” OR (behavio* W0 (counsel* or intervention* or train* or treatment* or therap* or psychotherap*)) OR (mood N2 monitor*)) ) | 27173 |
|  | S21 | TI ( (((operant or instrumental) W0 (conditioning or learning)) OR (“positive interaction*” or avoida* “coping or environmental contingenc*” or “contingency management”)) ) OR AB ( (((operant or instrumental) W0 (conditioning or learning)) OR (“positive interaction*” or avoida* “coping or environmental contingenc*” or “contingency management”)) ) | 1846 |
|  | S20 | TI ( (((positive N0 reinforc*) or (reinforc* N2 (environment* or experience*)) OR (behavio* N2 motivat*) or “motivational interviewing” OR (activit* N1 schedul*) OR ((pleas* or enjoyable or rewarding) N2 (activit* or event?))) ) OR AB ( (((positive N0 reinforc*) or (reinforc* N2 (environment* or experience*)) OR (behavio* N2 motivat*) or “motivational interviewing” OR (activit* N1 schedul*) OR ((pleas* or enjoyable or rewarding) N2 (activit* or event?))) ) | 9321 |
|  | S19 | TI ( (("behavio* activat*") OR ("behavio* activat*" or BATD) OR (behavio* N2 (reinforce* or "re inforce*" OR reinforce)) OR (behavio* N1 (contracting or modif*)) OR reinforc*) ) OR AB ( (("behavio* activat*") OR ("behavio* activat*" or BATD) OR (behavio* N2 (reinforce* or "re inforce*" OR reinforce)) OR (behavio* N1 (contracting or modif*)) OR reinforc*) ) | 32636 |
|  | S18 | (MH (“Behavior Therapy” OR “Motivational Interviewing”)) | 18301 |
|  | S17 | S15 AND S16 | 56277 |
|  | S16 | S6 OR S10 OR S13 OR S14 | 2399995 |
|  | S15 | ( (MH (Depression OR “Affective Disorders” OR “Dysthymic Disorder” OR “Cyclothymic Disorder”) ) OR TI ( (depress* or dysthymi* or cyclothymi* or “low mood” or “mood disorder*” or “affective disorder*”) ) OR AB ( (depress* or dysthymi* or cyclothymi* or “low mood” or “mood disorder*” or “affective disorder*”) ) | 233569 |
|  | S14 | MH (Neoplasms+) OR TI ( (cancer* or neoplas* or tumo* or carcinoma* or hodgkin* or nonhodgkin* or “non hodgkin*” or adenocarcinoma* or leukemia* or leukaemia* or metasta* or malignan* or lymphoma* or sarcoma* or melanoma* or myeloma* or oncolog* or psychooncolog* or glioma*) ) OR AB ( (cancer* or neoplas* or tumo* or carcinoma* or hodgkin* or nonhodgkin* or “non hodgkin*” or adenocarcinoma* or leukemia* or leukaemia* or metasta* or malignan* or lymphoma* or sarcoma* or melanoma* or myeloma* or o [...](javascript:showHistoryTerm('ctl00_ctl00_MainContentArea_MainContentArea_historyControl_HistoryRepeater_ctl13_ellipsis',true)) | 991757 |
|  | S13 | S11 OR S12 | 1128779 |
|  | S12 | TI ( (arteriosclero* or atherosclero* or “peripheral arter*” disease* or “blood pressure”) OR (cardio* or cardia* or cvd or heart* or coronary or angina or ventric* or myocard* or pericard* or ischem* or ischaem* or emboli* or cerebrovasc* or “cerebral vascular” or arrhythmi* or thrombo* or “atrial fibrillat*” or infarct* or apoplexy or stroke or strokes or poststroke or hypertensi*) ) OR AB ( (arteriosclero* or atherosclero* or “peripheral arter*” disease* or “blood pressure”) OR (cardio* or ca [...](javascript:showHistoryTerm('ctl00_ctl00_MainContentArea_MainContentArea_historyControl_HistoryRepeater_ctl15_ellipsis',true)) | 911639 |
|  | S11 | (MH (“Cardiovascular Diseases+” OR “Blood Pressure”) | 710068 |
|  | S10 | S7 OR S8 OR S9 | 316943 |
|  | S9 | TI ( ((HbA1c or A1C or A1c or Hb1c or ((glycated or glycosylated) W0 (hemoglobin or haemoglobin))) OR (NIDDM or T2D or T2DM or T1D or IDDM or MODY or T1DM)) ) OR AB ( ((HbA1c or A1C or A1c or Hb1c or ((glycated or glycosylated) W0 (hemoglobin or haemoglobin))) OR (NIDDM or T2D or T2DM or T1D or IDDM or MODY or T1DM)) ) | 42264 |
|  | S8 | TI ( (diabet* OR (noninsulin*-depend* or non-insulin*-depend* or noninsulin*depend* or non-insulin*depend*) OR (“fasting glucose” or “plasma glucose” or “glucose tolerance test*” or ((glycemic or glycaemic) N1 control*)) ) OR AB ( (diabet* OR (noninsulin*-depend* or non-insulin*-depend* or noninsulin*depend* or non-insulin*depend*) OR (“fasting glucose” or “plasma glucose” or “glucose tolerance test*” or ((glycemic or glycaemic) N1 control*)) ) | 259665 |
|  | S7 | (MH (“Diabetes Mellitus+” OR “Diabetes Mellitus, Type 2” OR “Diabetes Mellitus, Type 1” OR “Glucose Tolerance Test” OR “Blood Glucose”)) | 217401 |
|  | S6 | TI S1 OR S2 OR S3 OR S4 OR S5 | 284209 |
|  | S5 | TI ( (respirat* N1 (condition* or disease* or symptom* or problem* or failure or ill*)) ) OR AB ( (respirat* N1 (condition* or disease* or symptom* or problem* or failure or ill*)) ) OR TI ( (“pulmonary hypertension” OR COPD or COAD or COBD or AECB or AECOPD) ) OR AB ( (“pulmonary hypertension” OR COPD or COAD or COBD or AECB or AECOPD) ) | 55698 |
|  | S4 | TI ( ((“hyper responsiveness” or hyperresponsiveness or allergi* or hypersensitiv* or hyperreactiv* or insufficiency) N4 (airway or respirat* or bronchial* or lung*)) ) OR AB ( ((“hyper responsiveness” or hyperresponsiveness or allergi* or hypersensitiv* or hyperreactiv* or insufficiency) N4 (airway or respirat* or bronchial* or lung*)) ) OR TI ( ((longterm or “long term” or Chronic* or occupational or recur*) N1 lung* N4 (condition* or disease* or symptom* or problem* or failure* or ill*)) ) OR [...](javascript:showHistoryTerm('ctl00_ctl00_MainContentArea_MainContentArea_historyControl_HistoryRepeater_ctl23_ellipsis',true)) | 8013 |
|  | S3 | TI ( (((“long term” or longterm or chronic*) N4 (bronchitis or respirat*)) OR emphysema* OR asthma*) ) OR AB ( (((“long term” or longterm or chronic*) N4 (bronchitis or respirat*)) OR emphysema* OR asthma*) ) OR TI ( (obstruct* N2 (pulmonary or lung* or airway* or airflow* or bronch* or respirat*)) ) OR AB ( (obstruct* N2 (pulmonary or lung* or airway* or airflow* or bronch* or respirat*)) ) | 73564 |
|  | S2 | TI ( ((“non communicable” or noncommunicable or “non infectious” or noninfectious) N2 disease*) ) OR AB ( ((“non communicable” or noncommunicable or “non infectious” or noninfectious) N2 disease*) ) OR TI ( (chronic* N2 (condition or conditions or disease* or ill*)) ) OR AB ( (chronic* N2 (condition or conditions or disease* or ill*)) ) | 135272 |
|  | S1 | MH ((MH “Noncommunicable Diseases” OR “Chronic Disease” OR “Pulmonary Disease, Chronic Obstructive+” OR “Lung Diseases, Obstructive” OR “Asthma+” OR “Respiratory Hypersensitivity” Or “Hypertension, Pulmonary”)) | 144861 |
